# Evaluation of 1,2-Benzothiazine 1,1-Dioxide Derivatives In Vitro Activity towards Clinical-Relevant Microorganisms and Fibroblasts

**DOI:** 10.3390/molecules25153503

**Published:** 2020-07-31

**Authors:** Ruth K. Dudek-Wicher, Berenika M. Szczęśniak-Sięga, Rafał J. Wiglusz, Jan Janczak, Marzenna Bartoszewicz, Adam F. Junka

**Affiliations:** 1 Department of Pharmaceutical Microbiology and Parasitology, Faculty of Pharmacy, Medical University of Silesian Piasts in Wroclaw, Borowska 211, 50-556 Wroclaw, Poland; m.bartoszewicz@op.pl (M.B.); feliks.junka@gmail.com (A.F.J.); 2 Department of Medicinal Chemistry, Faculty of Pharmacy, Medical University of Silesian Piasts in Wroclaw, Borowska 211, 50-556 Wroclaw, Poland; berenika.szczesniak-siega@umed.wroc.pl; 3 Institute of Low Temperature and Structure Research, Polish Academy of Sciences, Okólna 2 str., P.O. Box 1410, 50-950 Wroclaw, Poland; r.wiglusz@intibs.pl (R.J.W.); j.janczak@intibs.pl (J.J.)

**Keywords:** 1,2-benzothiazines, antimicrobial activity, cytotoxicity, antiseptics, synthesis

## Abstract

The global concern related with growing number of bacterial pathogens, resistant to numerous antibiotics, prone scientific environment to search for new antimicrobials. Antiseptics appear to be suitable candidates as adjunctive agents to antibiotics or alternative local treatment option aiming to prevent and treat infections. 1,2-benzothiazines are considered one the most promising of them. In this research twenty 1,2-benzothiazine 1,1-dioxide derivatives were scrutinized with regard to their biological activity. Three of them are new. For evaluation of compounds’ activity against microbial pathogens, disk diffusion method and serial microdilution method was applied. To establish the cytotoxicity profile of tested 1,2-benzothiazines 1,1-dioxides derivatives, the cytotoxicity assay using fibroblasts L292 was performed. Antimicrobial activity of all tested compounds against Gram-positive *Staphylococcus aureus* and *Enterococcus faecalis* strains was higher than antimicrobial activity of DMSO solvent, which possesses antimicrobial activity itself. Gram-negative *P. aeruginosa*, *E. coli* and *K. pneumoniae* have shown susceptibility only to compounds **3e**, **7i** and **7l**. None of tested compounds was effective against *C. albicans.* Compound **6g** has demonstrated the strongest antimicrobial potency (MIC = 0.00975 mg/mL) among compounds of series **6**. Compounds of series **7**, namely **7d**, **7f**, **7g** had the lowest minimum inhibitory concentration (MIC). Compound **7f** displayed also the lowest cytotoxic effect against fibroblast cell line among series **7** compounds. All tested derivatives displayed lower MIC against Gram-positive bacteria than commercially applied antiseptic, povidone iodine, which MIC value range for tested Gram-positive bacteria was 1.56–6.25 mg/mL.

## 1. Introduction

The bacterial and fungal infections still pose a significant threat for patients’ health and life. One of the factors standing behind this dangerous phenomenon is microbial resistance to antibiotics. It is related not only with health complications, but also with significant economic burden resulting in therapeutic emergency experienced worldwide [1]. Bearing in mind a fact that microorganisms gain resistance in relatively short time, it is unlikely that recently approved antibiotics or these being in research pipeline would help to permanently stem aforementioned crisis [2]. Because of that, the use of non-antibiotic, antiseptic agents have skyrocketed in the past decades. However, there is increasing number of studies reporting that bacteria gain tolerance or resistance also to these substances [3,4].

Therefore, the increasing stress is put to development of novel approaches of infection treatment including a new, non-antibiotic, classes of antimicrobial agents. The heterocyclic thiazine and benzothiazine derivatives are important class of such compounds. They are biological constituents of numerous biomolecules and many of them are already known of their useful medicinal properties [5]. The nitrogen- and sulfur-containing benzothiazine derivatives possess the most comprehensive spectrum of biological activities. The biological and pharmacological properties of these compounds have been demonstrated already in a number of studies [6,7,8]. Depending upon the substituents, 1,2-benzothiazine derivatives have shown the specific biological activities, including antioxidant, anti-inflammatory and anticancer properties [6]. These compounds act as potent inhibitors of calpain I and may become potential drugs for neurodegenerative disorders including stroke, traumatic brain injury, spinal cord trauma, Alzheimer’s disease, Parkinson’s disease, multiple sclerosis and motor neuron damage [7]. Moreover, they elicit neuroprotective effects against 1-methyl-4-phenylpyridinium- (MPP+)-induced toxicity in human dopaminergic SH-SY5Y neuroblastoma cells [8].

**Scheme 1:**
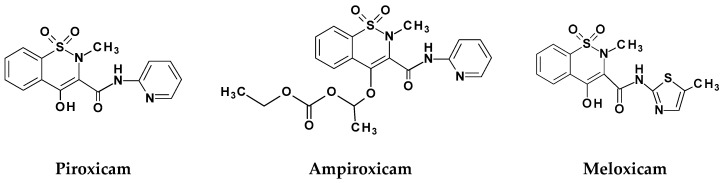
Structures of selected 1,2-benzothiazine-based drugs.

In recent years the significant interest in compounds containing the benzothiazine ring system (oxicams, the COX-1/2 inhibitors, mainly) was observed (Scheme 1) [9]. It has been shown that chemically modified 1,2-benzothiazine 1,1-dioxides are potent anti-inflammatory agents and their use does not correlate with ulcerogenicity occurrence [10]. In several studies, the chemopreventive, antioxidant and antimicrobial activity of the 1,2-benzothiazines was already shown [11,12,13]. In game-changing work by Soheili et al. it was suggested that 1,2-benzothiazine-based drugs can be developed and used as potential inhibitors of the *Pseudomonas aeruginosa*, one of the most resistant and common opportunistic pathogen in nosocomial setting [14]. 

Therefore, the aim of present research was to evaluate the in vitro antimicrobial potency and cytotoxicity of 20 compounds synthesized from 1,2-benzothiazine derivatives. Seventeen of them were synthesized by our team previously [10,15,16,17,18], and three of them were newly synthesized for the need of this research. However, none of them was previously scrutinized with regard to their antimicrobial activity and cytotoxicity. Additionally, to show the antimicrobial potential of studied compounds, two antiseptic agents—povidone-iodine (PVP-I) and polyhexanide (PHMB)—as a reference compounds were scrutinized. Presently, a variety of antiseptic solutions are used prior invasive procedures to remove potentially infective microorganisms from the skin. Among the antiseptics, povidone-iodine (PVP-I) is the most common, prevalent and widely-available antiseptic agent used in clinical practice [19]. It has well documented effectiveness and it is considered to have the broadest spectrum of antimicrobial action compared with other common antiseptics such as chlorhexidine, octenidine, polyhexanide and hexetidine [19]. In turn, polyhexanide is recommended for antisepsis of acute wounds. It seems to be preferable for chronic wounds thanks to its high tolerability. When a prolonged contact time is feasible, polyhexanide exhibits the strongest antimicrobial potency comparing to octenidine, chlorhexidine, triclosan and PVP-I [20].

## 2. Results

### 2.1. Chemistry

**Scheme 2:**
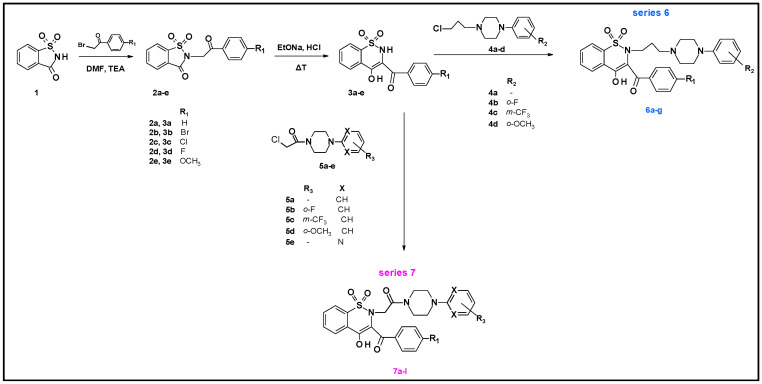
Synthetic route of series 6 and 7 compounds. TEA—triethylamine, DMF—dimethylformamide, EtONa—sodium ethanolate, HCl—hydrochloric acid. Structures of compounds are found in Tables 7 and 8.

Synthesis of compounds **3e**, **6a**–**6g** and **7a**–**7i** was done as previously reported [10,13,15,16,17]. Briefly, the key starting compound was commercially available saccharine 1 (Scheme 2). The reaction of 2-bromo-4′-methoxyacetophenone with saccharine in the presence of triethylamine (TEA) in dimethylformamide (DMF) afforded compound **2e**. Then Gabriel–Colman rearrangement of compound **2e**, through opening of a 1,2-thiazole ring in presence of sodium ethanolate and closing with formation of a 1,2-thiazine ring, resulted in compound **3e**. The next step was the reaction of key intermediates **3e** with appropriate 1-(2-chloro-1-oxoethyl)-4-arylpiperazine: **5a** for compound **7j**, **5e** for compound **7k** or **5d** for compound **7l** under reflux by 10 h. The scheduled new 1,2-benzothiazine derivatives **7j**, **7k** and **7l** received 37–51% yields. 

**Figure 1:**
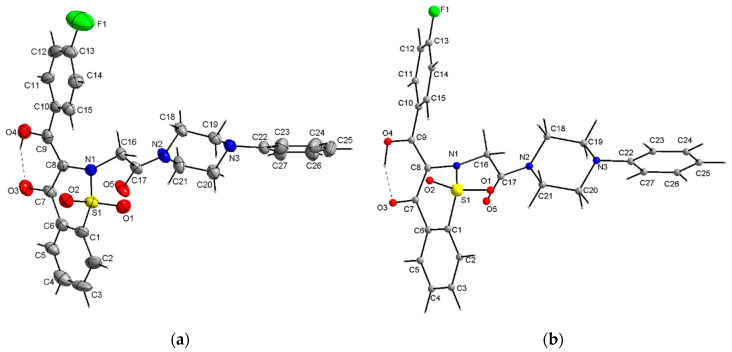
View of the X-ray molecular structure of **7i** showing displacement ellipsoids at the 30% probability level and H atoms as a sphere of arbitrary radii (**a**) and fully optimized DFT geometry (**b**). Dashed lines represent the intramolecular O-H···O hydrogen bonds.

The crystal structure of the new 1,2-benzothiazine derivatives was established by single crystal X-ray analysis of a representative compound **7i**. The compound **7i** crystallizes in the non-centrosymmetric space group *Fdd*2 of the orthorhombic system with 16 molecules in the unit cell. The asymmetric unit of the crystal consists of one molecule (Figure 1). The average S–O bond length of the SO_2_ group (1.420 A) as well as the carbonyl C17-O5 bond (1.219(6) Å) point on the double bond character (S=O) and the other C-C, C-N, C-O, C-S and S-N bond lengths are consistent with the distances observed in several of this type structures (Table 1). The C7-O3 bond with a distance of 1.275(6) Å is slightly longer carbonyl bond whereas the C9-O4 with a distance of 1.297(5) Å is slightly shorter the single C-O bond due to the partial delocalization of the π bond as a result of the formation of almost planar six membered ring with an O-H···O intramolecular hydrogen bond.

**Figure 2:**
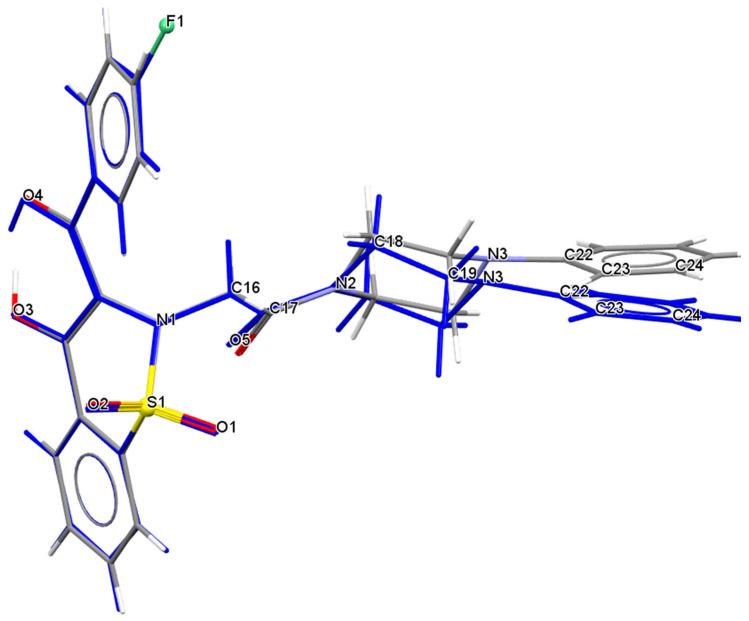
Comparison of conformation of whole molecule **7i** (marked in blue) with molecule **7a**.

Studies on the structure of piroxicam confirm the formation of such hydrogen bonds in this group of compounds [21] All phenyl rings in the molecule **7i** are planar, whereas the piperazine ring exhibits a chair conformation. The C1–C6 phenyl ring is almost coplanar with the C10–C16 one whereas the third C22–C27 phenyl ring is almost perpendicular to the both C1–C6 and C10–C16 phenyl rings. The conformation of whole molecule **7i** is slightly different to that of whole molecule **7a** (Table 2 and Figure 2) [10]. As can be seen from Figure 2 the conformation of a part of molecule consisting of thiazine ring together with both phenyl C1–C6 and C10–C15 rings is quite similar in both 1,2-benzothiazine derivatives **7i** and **7a**, while the conformation of the remained part of the molecules consisting of a piperazine ring linked to a benzene ring differs in these molecules.

**Figure 3:**
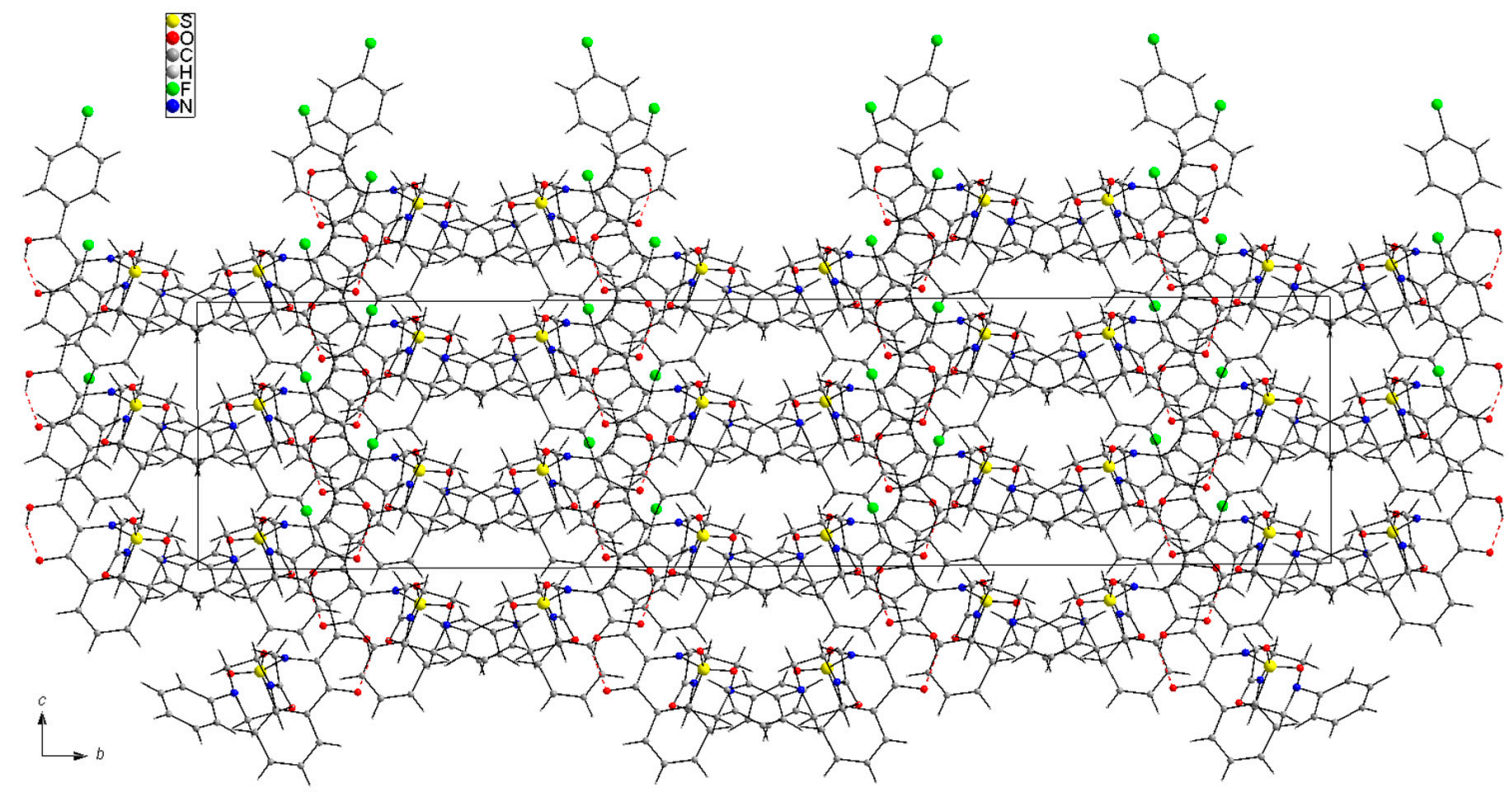
Arrangement of **7i** molecules in the unit cell viewed along a-axis. Dashed lines represent the intramolecular O-H···O hydrogen bonds.

The fully optimized molecule of **7i** performed by DFT (Density Functional Theory) calculation exhibits quite similar conformation as present in the crystal. Selected DFT geometrical parameters are listed together with the X-ray experimental values in the Table 1, whereas a full list of the DFT parameters are given in Supplementary Data (Table S1). The conformation of the whole molecule is stabilized by the intramolecular O3-H···O3 hydrogen bond (Table 3). Little differences between the X-ray and DFT conformations of the studied molecule **7i** results from a different approach to the description of the conformation of molecules, X-ray values refer to the conformation of molecules in crystals in which interactions between the molecules play a significant role and lead to crystallization and specific crystal packing, while DFT values refer to a single isolated molecule in the gaseous state omitting interactions between molecules, hence these differences. Arrangement of the **7i** molecules in the crystal is mainly determined by the electrostatic interaction and by the van der Waals forces, since there are no directional interactions (like as hydrogen bonds) between the molecules (Figure 3). 

The molecular electrostatic potential map (MESP) is related to the electronic density in a molecule and is a powerful tool for analyzing the interactions, both between the same molecules with each other and also is of great importance in forming guest-host complexes as is the case in biological systems [22,23,24].

**Figure 4:**
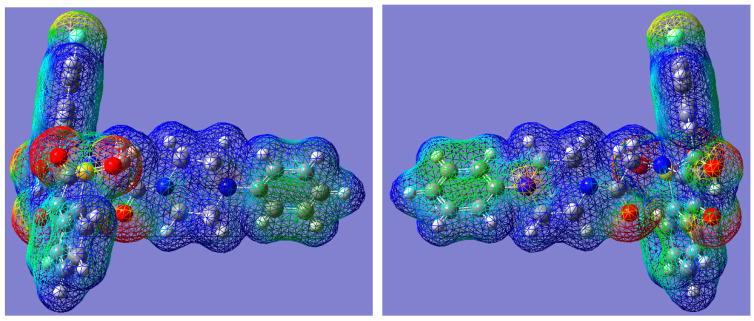
Three-dimensional molecular electrostatic potential map for **7i**. View from one side (**left**) and the other rotated by 180° (**right**). Color code: −0.05 eÅ^−1^ (red) to +0.05 eÅ^−1^ (blue).

The three-dimensional MESP map for **7i** was calculated on the basis of the DFT (B3LYP) optimized geometries of molecules and mapped onto the total electron density isosurface (0.008 eÅ^−3^) for both molecules using the GaussView 5.0 program (Figure 4). The color coding of MESP is in the range of –0.05 (red) to 0.05 eÅ^−1^ (blue). For compound **7i** the regions of negative MESP are usually associated with the lone pair of electronegative oxygen atoms of SO_2_ and carbonyl and hydroxyl groups, whereas the regions of positive MESP are associated with the electropositive atoms, mainly H atoms of aromatic rings and piperazine ring (Figure 4). Less negative MESP region is also associated near the N atoms. In addition, significantly less negative value of MESP than that near the oxygen atoms spreads across the aromatic planar phenyl rings. The three-dimensional MESP reflects the distribution of electron density within the molecule and is important in its biological activity as well as matching as a guest molecule in the active center of the biological system.

### 2.2. Biological Tests

In the agar disc diffusion method, 15 tested compounds out of 20 displayed antimicrobial activity against *S. aureus* strain. In case of compounds **6a**, **6c**, **6e**, **6f**, **7j** no inhibition zone was observed. Eleven compounds had antimicrobial activity against applied *E. faecalis* strain. Compounds **6a**, **6c**, **6e**, **6f**, **6g**, **7c**, **7h**, **7k**, **7l** have not inhibited E. *faecalis* growth. Compounds **6d**, **6g**, **7a**, **7b**, **7e**, **7f**, **7h**, **7l** were the most effective against *S. aureus*, while **6d**, **7b**, **7d**, **7f**, **7g**, **7j** against *E. faecalis*.

**Figure 5:**
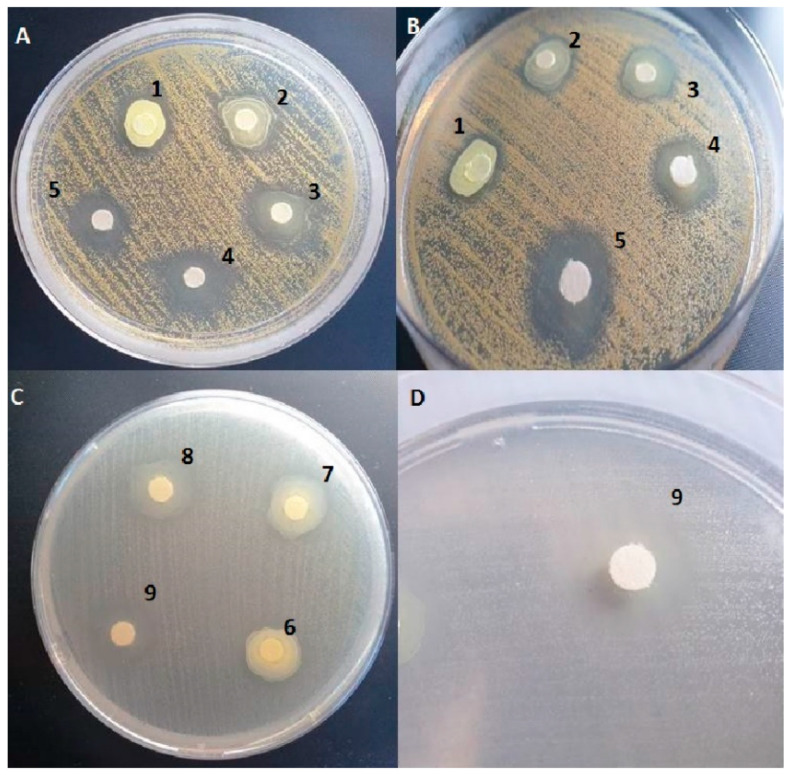
Inhibition zones of *S.aureus* in result of activity of compounds **6d** (**A**,**B**) activity. Inhibition zones of *E. faecalis* in result of activity of compounds **7f** (**C**,**D**) activity. Applied compounds’ quantities [mg/disc]: 1 = 0.5 mg; 2 = 0.25 mg; 3 = 0.125 mg; 4 = 0.0625 mg; 5 = 0.0312 mg; 6 = 0.25 mg; 7 = 0.125 mg; 8 = 0.0625 mg; 9 = 0.0312 mg.

None of the tested compounds was effective against *P. aeruginosa*, *K. pneumoniae*, *E. coli* and *C. albicans* within tested range of compounds’ applied quantities. Examples of growth inhibition zones are presented in Figure 5, while parametric results of selected 1,2-benzothiazine derivatives from this experiment are presented in Table 4. 

Activities of all tested compounds against *S. aureus* and *E. faecalis* are presented in Tables S2 and S3 respectively, as a diameter of inhibition zones [mm].

The concentrations of compounds were chosen with regard to the solubility of the compounds in dimethyl sulfoxide (DMSO). The highest tested concentration was at the same time the highest achievable through dissolution of the compound in DMSO. Thereafter, geometrical dilutions of initial concentrations have been tested.

In serial microdilution method all tested compounds were active against Gram-positive cocci within tested spectrum of concentrations (Table 5). The lowest MIC for *S. aureus* and E. *faecalis* was observed in case of compound **6b**, **6d**, **6e**, **6g**, **7d**, **7f**, and **7g**. In these cases, the lowest concentration of 0.097% DMSO was used. The compound **6g** was the most effective against *S. aureus* and E. *faecalis* with regard to MIC value obtained. Gram-negative *P. aeruginosa E. coli* and *K. pneumoniae* have shown susceptibility only to compounds **3e**, **7i** and **7l**. None of tested compounds was effective against *C. albicans*. 

The minimum inhibitory concentrations (MIC) of clinically used antiseptics: povidone-iodine (PVP-I) and polihexanidine (PHMB) are presented in Table 5. All tested derivatives had lower MIC (higher activity) against Gram-positive bacteria than PVP-I. Compounds **6b**, **6d**, **6e**, **7d**, **7f**, **7g** had the same MIC as PHMB against *S. aureus*. MIC of **6a**, **6b**, **6c**, **6d**, **6e**, **6g**, **7a**, **7d**, **7e**, **7f**, **7g** was lower against *E. faecalis* than MIC of PHMB. Compounds **3e**, **7i**, **7l**, active against Gram-negative bacteria, had lower MIC against *P. aeruginosa* than PHMB and PVP-I. Moreover, the aforementioned compounds displayed higher activity against *E. coli* and *K. pneumoniae* than PVP-I but not PHMB. 

Also, the Minimal Bactericidal Concentration (MBC) assay was performed for three compounds (**6d**, **6g**, **7f**), most active against Gram-positive pathogens and two compounds (**7l**, **7i**) most active against Gram-negative pathogens. For *S. aureus* the **6d** and **6g** MBC was 0.1 mg/mL and 0.625 mg/mL, respectively; no bactericidal activity was observed for these compounds within tested range of concentrations for *E. faecalis*. The MBC of **7i** and **7l** for all Gram-negative pathogens was equal 3.125 mg/mL (for details please refer to Tables S4 and S5 of Supplementary Materials). 

For cytotoxicity assay the compounds concentrations equal of MIC value were chosen. 

Seventeen compounds showed less than 50% level of cytotoxicity in cytotoxicity assay. The results of cytotoxicity of all tested compounds are presented in Table 6. The lowest cytotoxicity had been shown for derivatives **7a**, **7e**, **7f**, **7g**, **7j** and **7l** in both tested concentrations (Compound **7a** had low cytotoxicity in concentration 1 but in concentration 2, the cytotoxicity was 10 fold higher). The most cytotoxic were compounds **6d**, **3e** and **7i** in both concentrations. 

## 3. Discussion

Healthcare associated infections (HAIs) are the main cause of morbidity and mortality for individual patients and generate high economic burden for the healthcare system. Antiseptics have been demonstrated to be effective means to reduce the incidence of HAIs. Preoperative skin antisepsis using antiseptics is performed to reduce risk of surgical site infections (SSIs) and catheter-related infections (ICU), the third cause of infections in the intensive care units (ICU) [25]. However, concern has been raised in recent years for the emergence of reduced susceptibility with widespread use of these agents [26]. Thus, there is a need to search for new substances with antiseptic properties. Organic synthesis has focused on the modification of already existing compounds as well as on the development of novel molecules with novel sites of action. Prompted by the well-established antibacterial properties of benzothiazines, a series of 1,2-benzothiazine derivatives, covering different modifications of the benzothiazine scaffold, were synthesized as readily available starting materials for future drug candidates.

**Figure 6:**
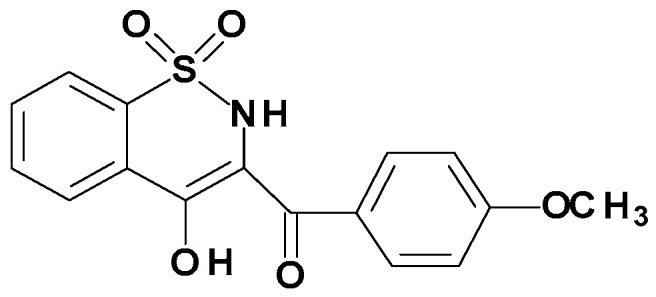
Chemical Structure of Compound **3e**.

1,2-benzothiazine 1,1-dioxide derivatives were assayed in vitro for the evaluation of their antimicrobial activity against Gram-positive *S. aureus* and *E. faecalis* and Gram-negative *P. aeruginosa*, *E. coli* and *K. pneumoniae* and antifungal properties against *C. albicans*. All studied compounds were divided into two series because of a type of a linker between thiazine and piperazine nitrogens. **Series 6** has propylene and **series 7** has 2-oxoethylene linker (Table 7 and Table 8). In order to compare the antimicrobial effects of an extended substituent on a thiazine nitrogen atom presented in compounds of **series 6** and **7**, compound **3e** denuded of such a substituent was also investigated (Figure 6). 

The four most active compounds described by Patel et al. were substituted with a halogen atom, and two of them, 38 and 43, had a halogen atom (Br, Cl) in the para position of the benzoyl moiety. It is consistent with the fact that all our most active compounds also have a halogen atom (Cl, F) and most of them in the para position of the benzoyl moiety (compounds **6b**, **6d**, **6e**, **7f** and **7g**) [11]. Comparing compound **6e**—one of the strongest (MIC = 0.024 mg/mL)—with a *p*-fluorobenzoyl substituent with compounds of the same propylene series without a fluorine substituent—**6a** or **6f**—they show weaker activity about 4–10 times (Table 5).

In **series 7**, the three strongest compounds—**7d, 7f** and **7g**—have one or two halogen atoms (Cl, F). The effect of halogen on **series 7** activity is clearly visible when comparing compounds **7b** and **7k**, which do not have a halogen atom, with compound **7e**, which has a p-bromobenzoyl substituent. Compound **7e** with MIC = 0.0655 mg/mL is about 2–3 times stronger than compounds **7b** and **7k** (Table 5).

Results of our study indicated that all scrutinized derivatives were active against Gram-positive bacteria, but **series 6**—propylene—were more active than **series 7**—2-oxoethylene (Table 5). For activity against Gram-positive bacteria, the presence of an extensive phenylpiperazine substituent in position two of the thiazine ring is favorable comparing to compound **3e** which does not have this substituent and thus it shows significantly weaker activity (Figure 6). Compounds **6b**, **6d**, **6e**, **6g**, **7d**, **7f** and **7g** of the highest activity against Gram-positive bacteria had in their structure a fluorine atom or trifluoromethyl group. The most active compound—**6g** with MIC = 0.00975 mg/mL—has in its structure *m*-CF_3_ group in phenylpiperazine moiety and *p*-methoxybenzoyl substituent in thiazine 3 position (Table 7).

Ahmad’s research on 1,2-benzothiazine-3-carbohydrazide 1,1-dioxides showed that compounds with greater lipophilicity possessed higher anti-bacterial activities [27]. In our research, propylene or 2-oxoethylene linkers have been introduced to the structure of 1,2-benzothiazine derivatives’ arylpiperazine moiety to increase the lipophilicity of the compounds, and in a consequence, to enhance their antibacterial activity.

Additionally, the type of the linker has an influence on the lipophilicity of new 1,2-benzothiazine derivatives thus **series 6** compounds with the propylene linker have slightly higher lipophilicity (logP = 2–3) than **series 7** compounds with 2-oxoethylene linker (logP = 1–2). The presence of different substituents in the phenyl ring of the benzoyl moiety also had an effect on the antibacterial activity against *S. aureus* and *E. faecalis.* Compounds with a halogen such as chlorine, fluorine or bromine atom in the *para* position of benzoyl moiety showed increased antibacterial activity. However compound **7d** does not have a halogen substituent in this position but its activity against Gram-positive organism was the same as those compounds which have these substituents. This may be caused by the presence of fluorine but in a different position—in a phenylpiperazine moiety.

Antimicrobial potential of tested derivatives was assessed using two methods. The agar diffusion method is not appropriate to determine the minimum inhibitory concentration (MIC) and cannot distinguish bactericidal and bacteriostatic effects of tested substances [28]. However, microdilution method was additionally included.

The results obtained for compounds **6g** and **7f** were coherent for agar diffusion method and microdilution method in case of activity against *S. aureus*. In case of *E. faecalis* the results obtained for compounds **6b**, **6d**, **7d** and **7f** were coherent for agar diffusion method and microdilution method (Figure 5, Table 4 and Table 5). Compound **7f** was active against *S. aureus* and *E. faecalis* in both methods. The observed discrepancy in results between agar disc diffusion method and MIC assessment (for example in case of compound **6e**, **7a**, **7g**, **7l**) may be caused by even such prosaic features as viscosity of specific derivatives’ suspension resulting in various area of agar covered with active compound. Moreover, the microbial cells spread on stable agar surface are less exposed to antimicrobial agent than cells suspended in fluid medium. We hypothesize that it may explain the observed discrepancies between results obtained by two testing methods. 

It should be also noted that in case of experimental compounds, the disc diffusion method is a preliminary screening technique. In this agar-based susceptibility testing method, the sizes of the zones of inhibition depend on two main variables, namely: susceptibility of the isolate and the diffusion rate of the drug through the agar medium. In turn, the latter-mentioned parameter consists of compound’s viscosity, density and hydrophilic/hydrophobic properties [29]. 

The interactions of the compounds with the agar medium during diffusion were observed by other research teams and this is particularly relevant to the diffusion of hydrophobic or amphipathic compounds [30].

Also, in our study it was observed that the release of the highest quantity of compounds was—seemingly surprising—correlated not only with the lowest inhibition zone but also with the formation of the precipitate around the disc (Table 4, Tables S2 and S3). To prove that growth of bacteria is inhibited also in the area where precipitate formed, an additional set of experiments was conducted (Figure S1). No visible growth of microorganisms was observed during 48 h of incubation proving the bactericidal activity within precipitant area.

It should be noted that the team of Valgas et al. already proved that such a precipitation of water–insoluble substances may hinder diffusion of antimicrobial substances into the agar [31]. Therefore, not always the highest concentration of introduced active compound correlates with the highest inhibition zone. As we already mentioned above, the disc diffusion method is considered a preliminary technique which is used as a navigation point for subsequently performed, more accurate MIC and MBC analyses. 

In microdilution method (MIC analysis) all tested compounds were active against Gram-positive bacteria (Table 5). This result stays in line with the results obtained by Patel et al. [11]. Compound **6g** was the most effective against *S. aureus* and *E. faecalis* and displayed acceptable (less than 50% of cell death) cytotoxicity of 21.24%. MIC of compound **6g** (0.00975 mg/mL) against *S. aureus* was lower than MIC of streptomycin (0.0125 mg/mL) which had been applied as a reference antibiotic in Platel’s investigation [11]. Compound **7f** had virtually lack of cytotoxicity (2.92%) and MIC equal 0.024 mg/mL. The compound **6g** had the strongest antimicrobial potential while compound **7f** displayed antimicrobial efficacy and the very low cytotoxicity. In a numerous research 1,2-benzothiazine derivatives were compared to such well-known antibiotics as imipenem, tetracycline, amoxicillin or above-mentioned streptomycin [6,32,33]. In our work different attitude is presented. As a reference, clinically used antiseptics of proven antimicrobial efficacy have been chosen. Our results indicate that compound **7f** was more active against Gram-positive bacteria than PVP-I and more active against *E. faecalis* than PHMB. It may suggest that this compound has antiseptic potential, which can be applied in clinical setting. 

Based on obtained results, certain correlation between compounds’ structure and cytotoxic profile may be hypothetically elucidated. Cytotoxicity analysis has shown that **series 7**-acetyl derivatives, are slightly less cytotoxic than **series 6**. Comparing **series 6** and **7** (Table 7 and Table 8) to compound **3e** (Figure 6) it can be seen that the expanded phenylpiperazine substituent at the 2-position of thiazine, regardless the type of linker, reduces the cytotoxicity of the compound. It may be concluded that different substituents in benzoyl and phenylpiperazine moiety have an impact on the cytotoxicity of new 1,2-benzothiazine derivatives. It seems that fluoro substituent in the *para* position of benzoyl moiety in a combination with *m*-trifluoromethyl group in phenylpiperazine increases the cytotoxicity of the compound (compound **6d** and **7h**). Whereas when aforementioned substituents are found singly or in combination with other substituents like Cl or OCH_3_ the cytotoxicity of compound decreased. The presence of *m*-trifluoromethyl group in phenylpiperazine, significantly enhances cytotoxicity when compared to compounds possessing *o*-methoxy substituent (compound **6d** vis **6a** and **6c**). Compounds which have no substituent in the benzoyl moiety (**6a**, **7a**, **7b**, **7c**, **7d**) stimulated fibroblasts’ growth with the exception of **7a**. This phenomenon highlights the great influence of substituent in this position on compound’s cytotoxicity. This finding is based on the observation that compound **7b**, without a substituent in benzoyl moiety stimulated fibroblasts’ growth, but its analog—**7e**—with a bromine atom in *para* position or **7k** with methoxy substituent did not. Likewise compounds **7c** and **7l** differ in the presence of a substituent in a benzoyl moiety. Both have a methoxy substituent in *ortho* position of phenylpiperazine moiety. However compound **7c** stimulated fibroblasts while **7l** did not. It suggests that the cytotoxicity of the 1,2-benzothiazine is more strongly affected by the substituent in benzoyl than phenylpiperazine moiety. 

Moreover, a fluorine atom in the *para* position of the benzoyl moiety significantly increased cytotoxicity (compound **7a** vis **7i**). Hence, it may be concluded that the substituent in the benzoyl moiety is important with regard to the cytotoxicity of the compound.

## 4. Materials and Methods 

### 4.1. Chemistry

All commercial chemicals were used as supplied unless otherwise indicated. 

^1^H and ^13^C-NMR spectra were recorded on a Bruker 300 MHz spectrometer (Bruker Polska Sp. z o.o. Poznan, Poland) using CDCl_3_ or DMSO-*d*_6_ as solvent. Chemical shifts for proton nuclear magnetic resonance (^1^H-NMR) spectra are reported in parts per million (ppm) relative to the signal of tetramethylsilane at 0 ppm (internal standard). Splitting patterns are designated as follows: s, singlet; brs, broad singlet; d, doublet; t, triplet; q, quartet; m, multiplet. Chemical shifts for carbon nuclear magnetic resonance (^13^C-NMR) spectra are reported in parts per million (ppm) relative to the center line of the CDCl_3_ triplet at 76.6 ppm. FT-IR spectra were recorded on a Perkin-Elmer Spectrum Two UATR FT-IR spectrometer (Perkinelmer Shared Services Sp. Z.o.o., Krakow, Poland). Mass data were acquired on a Bruker Daltonics micrOTOF-Q mass spectrometer (Bruker Polska Sp. z o.o. Poznan, Poland) in a positive ion mode with flow injection electron spray ionisation (ESI). The elemental analyses were performed by Carlo Erba NA 1500 analyser (Carlo Erba, Val de Reuil, France) and were within ±0.4% of the theoretical value. Melting points were determined in open glass capillaries using a MEL-TEMP melting-point apparatus (Electrothermal Engineering Ltd., Stone ST15 0SA, UK) and were uncorrected. Reaction progress was monitored by thin layer chromatography (TLC) performed on silica gel 60 F254 pre-coated aluminium sheets (Fluka Chemie GmbH, Buchs, Switzerland). Spots were visualized under 254 nm UV lamp. Rotary evaporator (Buchi Poland, Warszawa, Poland) under reduced pressure condition was used to concentrate the reaction solutions. Solvents were of reagent grade and purified with standard methods when necessary. 

### 4.2. General Procedure for the Preparation of Series ***6*** and Series ***7*** Compounds

Synthesis and experimental data of compounds **2a**–**e** and **3a**–**e** were previously reported in [10,15,18], those of **4a**–**d** and **5a**–**e** were reported in [15,16,17,34,35,36,37,38]. Synthesis and experimental data of compounds **6a**–**g** were described previously in [10,13,15,16,17].

#### Synthesis of **Series 7** Compounds (**7a**–**7l**):

5 mL of sodium ethanolate was added to the stirring solution of compound **3e** (5 mmol, 1.66 g) in 20 mL of anhydrous ethanol (EtOH). Next, 5 mmol of compound **5a** (for **7j**), **5e** (for compound **7k**) or **5d** (for compound **7l**) was added and refluxed with stirring for 10 h. Completion of the reaction was controlled using TLC plates. When the reaction was finished, ethanol (EtOH) was distilled off and remained residue was treated with 50 mL of chloroform (CHCl_3_), whereas insoluble compounds were filtered off. Afterwards, the filtrate was evaporated. To obtain the final products, the residue was purified by crystallization from EtOH. Compounds **7a**–**i** were synthesized in the manner previously described [10,13,16,17].

**Compound 7j—4-hydroxy-3-(4-methoxybenzoyl)-2-[2-(4-phenyl-1-piperazinyl)-2-oxoethyl]-2*H*-1,2-benzothiazine 1,1-dioxide** Yellow powder, 48% yield, mp 102–104 °C; FT-IR (cm^−1^): 1670, 1600 (C=O), 1345, 1180 (SO_2_). ^1^H-NMR (300 MHz, DMSO-*d*_6_) δ (ppm): 2.86–2.93 (m, 4H, H_piperazine_), 3.19–3.31 (m, 4H, H_piperazine_), 3.85 (s, 3H, OCH_3_), 4.12 (s, 2H, CH_2_CO), 6.81–6.84 (m, 3H, ArH, piperazine’s phenyl H), 7.13–7.19 (m, 4H, ArH, benzoyl group 2H and piperazine’s phenyl 2H), 7.84–7.88 (m, 3H, ArH, benzoyl group 2H and 1,2-benzothiazine’s 1H), 8.06–8.09 (m, 3H, ArH, 1,2-benzothiazine’s H), 15.23 (s, 1H, OH_enolic_). ^13^C-NMR (300 MHz, DMSO-*d*_6_) δ (ppm): 187.96, 168.17, 164.97, 163.59, 151.01, 138.77, 133.97, 133.05, 131.71, 129.40, 129.31, 127.87, 127.33, 122.28, 119.75, 116.57, 116.39, 116.22, 114.67, 56.05, 52.27, 48.55, 48.39, 44.53, 41.40. ESI-MS (*m/z*): 534.1683 [M + H]^+^; Anal. Calcd for C_28_H_27_N_3_O_6_S (533.16); C, 63.03; H, 5.10; N, 7.87; Found: C, 63.36; H, 5.35; N, 7.84.

**Compound 7k—4-hydroxy-3-(4-methoxybenzoyl)-2-{2-[4-(2-pyrimidyl)-1-piperazinyl]-2-oxoethyl}-2*H*-1,2-benzothiazine 1,1-dioxide** Yellow powder, 37% yield, mp 224–226 °C; FT-IR (cm^−1^): 1660, 1595 (C=O), 1340, 1170 (SO_2_). ^1^H-NMR (300 MHz, DMSO-*d*_6_) δ (ppm): 3.14 (brs, 4H, H_piperazine_), 3.48–3.55 (m, 4H, H_piperazine_), 3.86 (s, 3H, OCH_3_), 4.13 (s, 2H, CH_2_CO), 6.59–6.62 (t, 1H, *J* = 9.6 Hz ArH, CH(5)pyrimidine’s H), 7.13–7.16 (d, 2H, *J* = 9.0 Hz ArH, CH(4 and 6)pyrimidine’s H), 7.84–7.88 (m, 3H, ArH, benzoyl group H), 8.06–8.13 (m, 3H, ArH, benzoyl group 1H and 1,2-benzothiazine’s 2H), 8.31–8.32 (d, 2H, *J* = 4.8 Hz ArH, 1,2-benzothiazine’s H), 15.25 (s, 1H, OH_enolic_). ^13^C-NMR (300 MHz, DMSO-*d*_6_) δ (ppm): 187.82, 168.42, 165.25, 163.59, 161.39, 158.39, 138.83, 133.95, 133.04, 131.71, 129.38, 127.83, 127.31, 122.24, 116.53, 114.67, 110.90, 56.06, 52.38, 44.29, 43.34, 43.16, 41.28. ESI-MS (*m/z*): 536.1587 [M + H]^+^; Anal. Calcd for C_26_H_25_N_5_O_6_S (535.15); C, 58.31; H, 4.70; N, 13.08; Found: C, 57.90; H, 4.56; N, 12.68.

**Compound 7l—4-hydroxy-3-(4-methoxybenzoyl)-2-{2-[4-(2-methoxyphenyl)-1-piperazinyl]-2-oxoethyl}-2*H*-1,2-benzothiazine 1,1-dioxide** Yellow powder, 51% yield, mp 176–178 °C; FT-IR (cm^−1^): 1650, 1600 (C=O), 1350, 1175 (SO_2_). ^1^H-NMR (300 MHz, CDCl_3_) δ (ppm): 2.89 (brs, 4H, H_piperazine_), 3.33 (brs, 4H, H_piperazine_), 3.83 (s, 3H, OCH_3_ by phenylpiperazine), 3.90 (s, 3H, OCH_3_ by benzoyl), 4.37 (brs, 2H, CH_2_CO), 6.85–6.90 (m, 3H, ArH piperazine’s phenyl H), 7.00–7.03 (m, 3H, ArH, piperazine’s phenyl 1H and benzoyl group 2H), 7.74–7.77 (m, 2H, ArH, benzoyl group H), 7.84–7.87 (m, 1H, ArH, 1,2-benzothiazine’s H), 8.14–8.16 (m, 2H, ArH, 1,2-benzothiazine’s H), 8.24–8.26 (m, 1H, ArH, 1,2-benzothiazine’s H), 15.69 (s, 1H, OH_enolic_). ^13^C-NMR (300 MHz, CDCl_3_) δ (ppm): 187.78, 170.48, 164.36, 163.29, 152.19, 140.35, 138.70, 132.96, 132.36, 131.39, 129.63, 127.89, 127.54, 123.68, 122.11, 121.06, 118.44, 115.85, 114.08, 111.42, 55.42, 55.52, 51.63, 50.59, 50.12, 45.42, 41.89. ESI-MS (*m/z*): 564.1794 [M + H]^+^; Anal. Calcd for C_29_H_29_N_3_O_7_S (563.17); C, 61.80; H, 5.19; N, 7.46; Found: C, 61.82; H, 5.17; N, 7.39.

***Single Crystal X-ray Measurement.*** The single crystal of **7i** was used for data collection on a four-circle KUMA KM4 diffractometer (Kuma Diffraction Ltd., Wroclaw, Poland) equipped with two-dimensional CCD area detector at room temperature. The graphite monochromatized Mo-Kα radiation (λ = 0.71073 Å) and the ω-scan technique (Δω = 1°) were used for data collection. Data collection and reduction along with absorption correction were performed using *CrysAlis* software package [39]. The structures were solved by direct methods using *SHELXT* [40] giving positions of almost all non-hydrogen atoms. The structures were refined using *SHELXL-2018* [41] with the anisotropic thermal displacement parameters. Hydrogen atoms were refined as rigid. Details of the data collection parameters, crystallographic data and final agreement parameters are collected in Table S6. Visualizations of the structures were made with the Diamond 3.0 program [42]. The structure has been deposited with the Cambridge Crystallographic Data Center in the CIF format, no. CCDC 2,008,685 for **7i**. Copies of this information can be obtained free of charge from The Director, CCDC, 12 Union Road, Cambridge, CB2 1EZ, UK (fax: +44 1223 336 033); email: deposit@ccdc.cam.ac.uk or www: http://www.ccdc.cam.ac.uk.

***DFT calculations***. Molecular orbital calculations with full geometry optimization of **7i** was performed with the Gaussian09 program package [43]. All calculations were carried out with the DFT level using the Becke3-Lee–Yang–Parr correlation functional (B3LYP) [44,45,46,47] with the 6-31+G basis set assuming the geometry resulting from the X-ray diffraction study as the starting structure. As convergence criteria, the threshold limits of 0.00025 and 0.0012 a.u. were applied for the maximum force and the displacement, respectively. The three-dimensional molecular electrostatic potential (3D MESP) maps are obtained on the basis of the DFT (B3LYP/6-31G) optimized. The calculated 3D MESP is mapped onto the total electron density isosurface (0.008 eÅ^−3^) for each molecule. 

### 4.3. Biological Tests

(a) the five following bacterial strains and one fungal strain from the American Tissue and Cell Culture Collection (***ATCC***) were applied in this study: *Staphylococcus aureus* 6538; *Pseudomonas aeruginosa* 15442, *Enterococcus faecalis* 29212, *Klebsiella pneumoniae* 70063, *Escherichia coli* 2592, *Candida albicans* 10231.
(b) fibroblasts L929 (***ATCC^®^ CCL-1***) were used to evaluate cytotoxic potential of the tested compounds.
(c) all microbial strains and fibroblast line are part of Strain and Line Collection of Pharmaceutical Microbiology and Parasitology Department of Medical University of Wroclaw
(d) 1,2-benzothiazine 1,1-dioxide derivatives were synthesized in the Department of Chemistry of Drugs of Medical University of Wroclaw. All tested compounds were divided to three series. Series 6 includes seven compounds, series 7 includes 12 compounds and series 3 is represented by one compound. All tested compounds are presented in the Figure 6, Table 7 and Table 8 below.

#### 4.3.1. Evaluation of the Antimicrobial Activity of 1,2-Benzothiazine 1,1-Dioxide Derivatives Using Disc Diffusion Method

All tested compounds (**1**–**20**) were dissolved in 100% dimethyl sulfoxide (DMSO, Stanlab, Lublin, Poland). Paper discs (diameter 5 mm) were placed onto the surface of the M-H agar medium (BioMaxima, Lublin, Poland) seeded with the suspension of aforementioned strains at a density of 0.5 McFarland using a densitometer (Biomerieux, Warszawa, Poland). Next, 20 µL of each quantity of 20 tested derivatives was poured on the paper discs. As a control, 20 µL of 100% DMSO was used. Subsequently, the cultures were carried out at 37 °C for 24 h. The results were presented as diameter of growth inhibition zone [mm]. The tests were performed in triplicates.

#### 4.3.2. Evaluation of the Minimum Inhibitory Concentration (MIC) of 1,2-Benzothiazine 1,1-Dioxide Derivatives vs. Povidone-Iodine (PVP-I) and Polihexanidine (PHMB) Antiseptics Using Serial Microdilution Method

The standard serial microdilution method, performed in a 96-well plate, was applied to compare the activity of analyzed derivatives against *S. aureus*, *P. aeruginosa*, *E. faecalis*, *K. pneumoniae*, *E.coli* and *C. albicans*. The derivatives’ antimicrobial activity was compared with activity of clinically-used antiseptics, namely povidone-iodine (later referred to as the PVP-I; Braunol, Braun) and polyhexanide (later referred to as the PHMB, Prontosan, Braun). 

The cultures of microbial strains growing on agar plates were transferred to liquid Tryptone Soya Broth (TSB, BTL) and incubated at 37 °C for 24 h. Next, optical density of 1 McFarland was settled using a densitometer (Biomerieux, Poland). And the suspension was diluted in the Miller-Hinton Broth (M-H, BioMaxima, Poland) medium to reach the density of 1 × 10^5^ cells/mL. 

Afterwards, 1,2-benzothiazine 1,1-dioxide derivatives were dissolved in 100% dimethyl sulfoxide (DMSO, Stanlab, Poland). Serial dilutions of each compound, PVP-I and PHMB were performed with M-H Broth. Next 100 μL of previously-prepared microbial suspension was added to each well. There was 2-fold dilution of compound used in each subsequent well. The highest tested concentration of each 1,2-benzothiazine derivative is shown in Table S7 of Supplementary Materials. Plates were incubated for 24 h/37 °C. Afterwards, 20 μL of 0.1% triphenyltetrazolium chloride (TTC, Sigma-Aldrich, Poznan, Poland) was added to the wells. Reduction of colorless TTC to red formazan confirmed the presence of metabolically active microorganisms in the plate’s well. The first colorless well of the plate showed compound’s or antiseptic’s MIC. In order to ensure that the DMSO solvent had no effect on bacterial growth, a control test was performed. For this purpose, geometric dilutions of DMSO in M-H broth were made and 100 μL of microbial suspension 1 × 10^5^ cells/mL was added to each well. The sterility control setting was M-H broth with no microorganisms added; the growth control setting was M-H broth with microbial suspension, but no antimicrobial compound added. All procedures were performed in triplicates. To additionally check correctness of TTC-based MIC assay, the microbial turbidity studies were performed using Thermo Scientific Multiskan Go spectrometer (Thermo-Fischer Scientific, Vantaa, Finland) with wavelength of 580nm for three compounds (**6d**, **6g**, **7f**) most active against Gram-positive pathogens and two compounds (**7l**, **7i**) most active against Gram-negative pathogens. Next, the standard procedure of MBC evaluation according to CLSI (Methods for Determining Bactericidal Activity of Antimicrobial Agents; Approved Guideline, document M26A, https://clsi.org) was performed.

#### 4.3.3. Cytotoxicity Assay on L929 Fibroblast Cells

The standard neutral red cytotoxicity test was performed. A 100 μL of suspension of fibroblasts L929 of 10^5^ cells/mL density was added to 96-well plate and incubated for 24 h at 37 °C. After incubation, the medium was removed and MIC concentrations of tested derivatives were poured into the wells (100 μL/well). Concentrations of tested derivatives selected for cytotoxicity assay contains Table 6. Three wells were the positive growth control in the culture medium DMEM (Dulbecco’s Modified Eagle’s Medium) with high glucose content (4.5 g/L), with the addition of 1% penicillin, 1% amphotericin and 2% fetal serum. The cultures were carried out at 37 °C for 24 h. Next, a solution of neutral red (NR) was prepared by adding 1 mL of NR (0.33%, Sigma Poland) to 0.6 mL of deionized water. Afterwards, 0.5 mL of this mixture was added to 39.5 mL of culture medium to achieve proper dilution of NR. 100 μL of neutral red medium was introduced to wells of the plate. Cell with dye-containing medium were incubated three hours at 37 °C. After incubation, the neutral red solution was aspirated off and the plates were desiccated. Then, 100 µL of de-satin solution (50% ethanol, 49% deionized water and 1% glacial acetic acid) was introduced to each well. The plates were shaken in a microtiter plate shaker for 30 min. until neutral red has been extracted from the cells. The value of neutral red absorbance was measured in 540 nm wavelength using Thermo Scientific Multiskan Go spectrometer (Thermo-Fischer Scientific, Vantaa, Finland). 

The absorbance was calculated as % cytotoxicity (%C_tx_) using the formula:

%C_tx_ = 100% − (A_b_/A_k_) × 100% (1)

A_b_ = Absorbance of the sample

A_k_ = Absorbance of control 

The absorbance values of samples and control are presented in Supplement Materials (Table S8). The absorbance value of cells untreated with tested derivatives was considered 100% of potential cellular growth (positive control). All compounds tested were DMSO-solved. Concentrations of DMSO used in case of particular 1,2-benzothiazine derivatives are shown in Table S9 of Supplementary Data. Because specific concentrations of DMSO are considered cytotoxic, additional control sample was performed. It consisted of cells incubated in medium containing different concentration of DMSO. Cytotoxicity of applied concentrations of DMSO is reported in Supplementary Data (Table S10).

## 5. Conclusions 

To summarize, all tested 1,2-benzothiazine 1,1-dioxide derivatives were active against Gram-positive cocci. The most effective among compounds of series **6** was compound **6g**. Among derivatives of series **7**, compound **7f** had low MIC and lack of cytotoxic properties. In conclusion, the results of this study revealed that 1,2-benzothiazine 1,1-dioxide derivatives could be potential antimicrobials and may become a useful template for future drug development through modification or derivatization to design more potent biologically active compounds [5]. 

## Figures and Tables

**Table 1:** Selected geometrical parameters (Å, °) for **7i**.

	X-ray	DFT
S1—O1	1.420 (3)	1.468
S1—O2	1.420 (4)	1.467
S1—N1	1.618 (4)	1.684
S1—C1	1.742 (6)	1.790
C7—O3	1.275 (6)	1.254
C8—N1	1.429 (6)	1.433
C9—O4	1.297 (5)	1.331
N1—C16	1.477 (6)	1.462
C17—O5	1.219 (6)	1.229
C17—N2	1.318 (6)	1.368
N3—C22	1.427 (6)	1.420
O2—S1—O1	119.3 (2)	120.18
N1—S1—C1	102.4 (3)	101.34
C5—C6—C7—C8	−170.0 (6)	−169.97
C8—C9—C10—C15	−30.0 (8)	−39.05
C8—N1—C16—C17	−69.0 (5)	−62.23
S1—N1—C16—C17	85.8 (5)	92.72
N1—C16—C17—N2	178.1 (4)	178.14
C19—N3—C22—C23	7.3 (5)	10.27

**Table 2:** Comparison of the selected X-ray conformational parameters (Å, °) for **7i** and **7a**.

	7i	7a
C1—S1—N1—C16	−106.8 (4)	−107.7 (4)
S1—N1—C16—C17	85.7 (4)	82.7 (4)
N1—C16—C17—O5	4.0 (6)	0.3 (7)
N1—C16—C17—N2	178.1 (4)	−178.9 (5)
C16—C17—N2—C18	−1.4 (7)	−7.1 (7)
C17—N2—C18—c19	129.8 (5)	139.7 (7)
N2—C18—C19—N3	58.5 (6)	57.7 (6)
C18—C19—N3—C22	166.5 (5)	165.0 (5)
C19—N3—C22—C23	7.3 (7)	15.5 (7)
N3—C22—C23—C24	−174.9 (5)	−176.6 (5)

**Table 3:** Hydrogen-bond geometry (Å, °).

*D*-H···*A*	*D*-H	H···*A*	*D*···*A*	*D*-H···*A*	
O4-H4A···O3	0.82	1.69	2.433 (6)	150	(in crystal)
O4-H4A···O3	1.011	1.599	2.519	148.90	(DFT)

**Table 4:** Mean diameter of inhibition zones obtained by loaded discs with various compounds’ quantities of tested 1,2-benzothiazine derivatives. Values in brackets are standard errors of mean.

Zones of *S. aureus* Growth Inhibition [mm]												
	4 mg	2 mg	1 mg	0.5 mg	0.25 mg	0.2 mg	0.125 mg	0.1 mg	0.0625 mg	0.05 mg	0.0312 mg	0.025 mg
**6d**	0	0	8 (±0)	8 (±0)	8 (±0)	N/A	9 (±0)	N/A	9 (±0)	N/A	10 (±0)	N/A
**6g**	N/A	N/A	N/A	N/A	N/A	6 (±0)	N/A	6 (±0)	N/A	8 (±0)	N/A	9 (±0)
**7a**	0	0	9.33 (±0.58)	9.66 (±1.15)	8.33 (±0.58)	N/A	9 (±1)	N/A	0	N/A	N/A	N/A
**7b**	10 (±0)	10 (±0)	10.33 (±0.58)	10 (±0)	9.33 (±0.58)	N/A	7.33 (±1.15)	N/A	0	N/A	N/A	N/A
**7e**	0	0	0	9 (±0)	9 (±0)	N/A	8 (±0)	N/A	6 (±0)	N/A	N/A	N/A
**7f**	0	0	0	9 (±0)	8 (±0)	N/A	9 (±0)	N/A	10 (±0)	N/A	10 (±0)	N/A
**7h**	0	10 (±0)	8 (±0)	9 (±0)	9 (±0)	N/A	0	N/A	0	N/A	N/A	N/A
**7l**	0	0	9 (±0)	9 (±0)	8 (±0)	N/A	9 (±0)	N/A	0	N/A	N/A	N/A
**Zones of *E. faecalis* Growth Inhibition [mm]**												
**6d**	0	0	0	10 (±0)	10 (±0)	N/A	11 (±0)	N/A	10 (±0)	N/A	10.33 (±0.58)	N/A
**7b**	0	0	0	9 (±0)	8 (±0)	N/A	8 (±0)	N/A	0	N/A	N/A	N/A
**7d**	0	0	0	8 (±0)	7 (±0)	N/A	7 (±0)	N/A	N/A	N/A	N/A	N/A
**7f**	0	0	0	6 (±0)	8 (±0)	N/A	10 (±0)	N/A	11 (±0)	N/A	11.33 (±0.58)	N/A
**7g**	0	0	0	0	9 (±0)	N/A	10 (±0)	N/A	8 (±0)	N/A	N/A	N/A
**7j**	0	0	0	9 (±0)	9 (±0)	N/A	9 (±0)	N/A	6 (±0)	N/A	N/A	N/A

N/A—Not applicable.

**Table 5:** In vitro antimicrobial activity [MIC [mg/mL] of tested compounds vs. clinically used antiseptics (PHMB—polyhexanide; PVP-I—povidone-iodine).

MIC [mg/mL]					
	*S. aureus*	*E. faecalis*	*P. aeruginosa*	*E. coli*	*K. pneumoniae*
**3e**	1.04	2.08	**1.04**	**1.04**	**1.04**
**Series 6**					
**6a**	0.078	0.078	2.5	1.25	1.25
**6b**	**0.024**	**0.024**	3.125	3.125	3.125
**6c**	0.065	0.065	2.07	2.07	2.07
**6d**	**0.024**	**0.024**	3.125	3.125	3.125
**6e**	**0.024**	**0.024**	3.125	3.125	3.125
**6f**	0.195	0.195	3.125	3.125	3.125
**6g**	**0.00975**	**0.00975**	1.25	1.25	1.25
**Series 7**					
**7a**	0.26	0.065	4.16	4.16	4.16
**7b**	0.195	0.39	6.25	6.25	6.25
**7c**	0.13	0.13	2.07	2.07	2.07
**7d**	**0.024**	**0.024**	3.125	3.125	3.125
**7e**	0.0655	0.0655	0.525	0.525	0.525
**7f**	**0.024**	**0.024**	3.125	3.125	3.125
**7g**	**0.024**	**0.024**	3.125	3.125	3.125
**7h**	0.78	0.78	3.125	3.125	3.125
**7i**	0.78	0.78	**1.56**	**1.56**	**1.56**
**7j**	0.195	0.39	3.125	3.125	3.125
**7k**	0.156	0.156	0.625	0.625	0.625
**7l**	0.195	0.39	**1.56**	**3.125**	**3.125**
**Antiseptics**					
**PHMB**	0.024	0.097	3.125	0.097	0.39
**PVP-I**	1.56	6.25	12.5	6.25	3.125

**Table 6:** Cytotoxicity of MIC of 1,2-benzothiazine derivatives.

	MIC [mg/mL]	Cytotoxicity [%]
**3e**	1.04 *^,^** 2.08 ^#^	59.55 (±12.39) 66.32 (±5.44)
**Series 6**		
**6a**	0.078 *^,#^	−1.51 (±5.30)
**6b**	0.024 *^,#^	15.11 (±1.19)
**6c**	0.065 *^,#^	15.61 (±7.80)
**6d**	0.024 *^,#^	79.76 (±0.62)
**6e**	0.024 *^,#^	23.61 (±5.21)
**6f**	0.195 *^,#^	15.10 (±2.69)
**6g**	0.00975 *^,#^	21.25 (±6.26)
**Series 7**		
**7a**	0.26 * 0.065 ^#^	2.40 (±14.82) 20.97 (±6.99)
**7b**	0.195 * 0.39 ^#^	−10.83 (±5.19) −3.795 (±6.94)
**7c**	0.13 *^,#^	−2.32 (1.43)
**7d**	0.024 *^,#^	−11.09 (±1.96)
**7e**	0.0655 *^,#^	2.68 (±4.20)
**7f**	0.024 *^,#^	2.92 (±13.42)
**7g**	0.024 *^,#^	9.68 (±4.09)
**7h**	0.78 *^,#^	38.26 (±10.86)
**7i**	0.78 *^,#^ 1.56 **	65.60 (±1.55) 61.64 (±4.98)
**7j**	0.195 * 0.39 ^#^	4.30 (±6.19) 32.53 (±0.67)
**7k**	0.156 *^,#^	17.27 (±13.97)
**7l**	0.195 * 0.39 ^#^	5.90 (±3.49) 3.11 (±2.30)

*: MIC against *S. aureus*, #: MIC against *E. faecalis*, **: MIC against Gram-negative bacteria.

**Table 7:** Structures of **series 6** compounds.

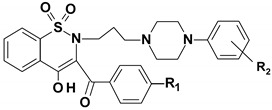 Series 6		
Compound	R_1_	R_2_
**6a**	H	*o*-OCH_3_
**6b**	Cl	*o*-F
**6c**	F	*o*-OCH_3_
**6d**	F	*m*-CF_3_
**6e**	F	-
**6f**	OCH_3_	-
**6g**	OCH_3_	*m*-CF_3_

**Table 8:** Structures of **series 7** compounds.

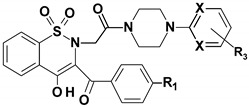 Series 7			
Compound	R_1_	R_3_	X
**7a**	H	-	CH
**7b**	H	-	N
**7c**	H	*o*-OCH_3_	CH
**7d**	H	*o*-F	CH
**7e**	Br	-	N
**7f**	Cl	*o*-F	CH
**7g**	F	*o*-F	CH
**7h**	F	*m*-CF_3_	CH
**7i**	F	-	CH
**7j**	OCH_3_	-	CH
**7k**	OCH_3_	*-*	N
**7l**	OCH_3_	*o*-OCH_3_	CH

## Supplementary Materials

Table S1. DFT optimized parameters of **7i.** Spectroscopic data for compounds **7j**, **7k** and **7l**. Table S2. Inhibition zones [mm] of *S. aureus* growth being result of tested compounds’ activity. Table S3. Inhibition zones [mm] of *E. faecalis* growth being result of tested compounds’ activity. Table S4. MIC and MBC values (mg/mL) for Gram-positive microorganisms. “X”—no bactericidal activity (MBC) in tested concentration of compound applied. Table S5. MIC and MBC values for Gram-negative microorganisms. “X”—no bactericidal activity (MBC) in tested concentration of compound applied. Table S6. Crystal data and details of the structure determination for compound **7i**. Table S7. The highest tested concentration 1,2-benzothiazine 1,1-dioxide derivatives. Table S8. The absorbance values of samples and control. Table S9. Concentrations of DMSO [%] used for 1,2-benzothiazine 1,1-dioxide derivatives dissolution to perform cytotoxicity assay. Table S10. Cytotoxicity of applied concentrations of DMSO. The cytotoxicity was calculated as % (%C_tx_) using the formula: %C_tx_ = 100% − (A_b_/A_k_ )× 100%. Figure S1. Inhibition of bacterial growth in the area of precipitation zone.
