# Supplementary material for: Evaluation of 1,2-Benzothiazine 1,1-Dioxide Derivatives In Vitro Activity towards Clinical-Relevant Microorganisms and Fibroblasts
Source: Molecules. 2020 Jul 31;25(15):3503. doi: 10.3390/molecules25153503 (PMC7435855; doi:10.3390/molecules25153503)
Supplement: Supplementary file 1 [file molecules-25-03503-s001.pdf]

Supplementary materials

Evaluation of 1,2 - Benzothiazine 1,1-Dioxide Derivatives *In Vitro* Activity Towards Clinical-Relevant Microorganisms and Fibroblasts

Ruth K. Dudek-Wicher [1]*, Berenika M. Szczęśniak-Sięga[2], Rafał J. Wiglusz[3], Jan Janczak[3], Marzenna Bartoszewicz[1] and Adam F. Junka [1]

[1] Department of Pharmaceutical Microbiology and Parasitology, Faculty of Pharmacy, Medical University of Silesian Piasts in Wroclaw; r.dudek.wicher@gmail.com; m.bartoszewicz@op.pl; feliks.junka@gmail.com

[2] Department of Medicinal Chemistry, Faculty of Pharmacy, Medical University of Silesian Piasts in Wroclaw; berenika.szczesniak-siega@umed.wroc.pl

[3] Institute of Low Temperature and Structure Research, Polish Academy of Sciences, Okólna 2 str., P.O. Box 1410, 50-950 Wroclaw, Poland; r.wiglusz@intibs.pl; j.janczak@intibs.pl*

Correspondence: r.dudek.wicher@gmail.com; Tel +48-606-763-589

Table S1. DFT optimized parameters of **7i.**

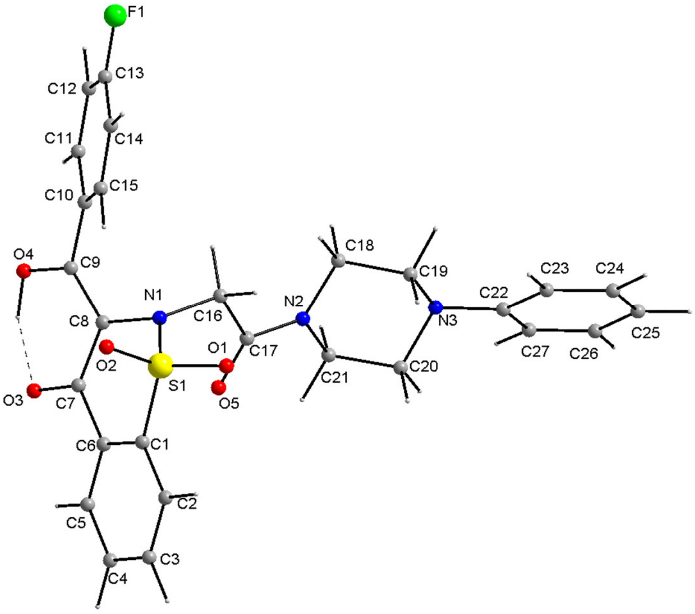

S1–O1	1.468	S1–O2	1.467	S1–N1	1.684
S1–C1	1.790	C1–C2	1.382	C2–C3	1.387
C3–C4	1.399	C4–C5	1.394	C5–C6	1.401
C6–C7	1.493	C7–O3	1.254	C7–C8	1.456
C8–N1	1.433	C8–C9	1.388	C9–O4	1.331
C9–C10	1.478	C10–C11	1.407	C11–C12	1.394
C12–C13	1.390	C13–F1	1.356	C13–C14	1.391
C14–C15	1.394	N1–C16	1.462	C16–C17	1.541
C17–O5	1.229	C17–N2	1.368	N2–C18	1.462
C18–C19	1.534	C19–N3	1.458	N3–C20	1.471
C20–C21	1.527	C21–N2	1.465	N3–C22	1.420
C22–C23	1.406	C23–C24	1.399	C24–C25	1.394
C25–C26	1.400	C26–C27	1.392	C27–C22	1.410
C_{ar}–H	1.087	C–H	1.091	O4–H	1.011
O1–S1–O2	120.18	N1–S1–O1	107.45	N1–S1–O2	107.68
N1–S1–C1	101.34	C1–C2–C3	119.91	C2–C3–C4	120.09

C3–C4–C5	120.34	C4–C5–C6	120.57	C5–C6–C7	119.02
C6–C7–C8	119.91	C6–C7–O3	118.85	C7–C8–C9	120.01
C8–C9–O4	120.69	C8–C9–C10	125.87	C7–C8–N1	119.68
N1–C8–C9	120.30	C9–C10–C11	118.88	C10–C11–C12	120.79
C11–C12–C13	118.32	C12–C13–C14	122.56	C12–C13–F1	118.70
C13–C14–C15	118.64	N1–C16–C17	114.17	C16–C17–O5	120.64
C16–C17–N2	116.82	C17–N2–C18	126.24	N2–C18–C19	110.94
C18–C19–N3	110.40	C19–N3–C20	111.13	N3–C20–C21	110.82
C20–C21–N2	110.50	C18–N2–C21	113.70	C19–N3–C22	117.71
N3–C22–C23	122.65	C22–C23–C24	120.67	C23–C24–C25	120.81
C24–C25–C26	118.87	C25–C26–C27	120.70	C26–C27–C22	120.91

N1–S1–C1–C2	151.68	S1–C1–C2–C3	177.71
C1–C2–C3–C4	-0.26	C2–C3–C4–C5	0.35
C3–C4–C5–C6	0.08	C4–C5–C6–C7	0.01
S1–C1–C6–C7	2.06	C5–C6–C7–C8	-169.96
C5–C6–C7–O3	13.77	C6–C7–C8–C9	-166.83
C7–C8–C9–O4	-9.81	O4–C9–C8–N1	170.11
C8–N1–S1–O1	167.53	C7–C8–C9–C10	171.71
C8–C9–C10–C11	144.84	C9–C10–C11–C12	178.02
C10–C11–C12–C13	-1.22	C11–C12–C13–C14	-0.18
C12–C13–C14–C15	0.98	C8–N1–C16–C17	-62.23
N1–C16–C17–O5	-1.72	N1–C16–C17–N2	178.14
C16–C17–N2–C18	-2.23	C17–N2–C18–C19	132.54
N2–C18–C19–N3	54.70	C18–C19–N3–C20	-58.04
C19–N3–C20–C21	58.34	N3–C20–C21–N2	-54.57
C18–C19–N3–C22	52.95	C19–N3–C22–C23	10.26
N3–C22–C23–C24	-177.81	C22–C23–C24–C25	-0.73
C23–C24–C25–C26	0.47	C24–C25–C26–C27	0.23
C25–C26–C27–C22	0.67		

Spectroscopic data for compounds 7j, 7k and 7l

^{1}H NMR and ^{13}C NMR spectra

Compound	Structure	Page
**7j**	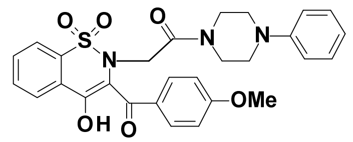	S3
**7k**	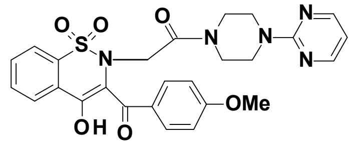	S4
**7l**	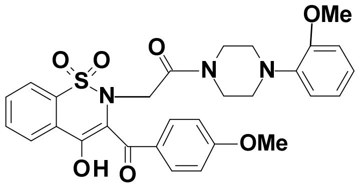	S5

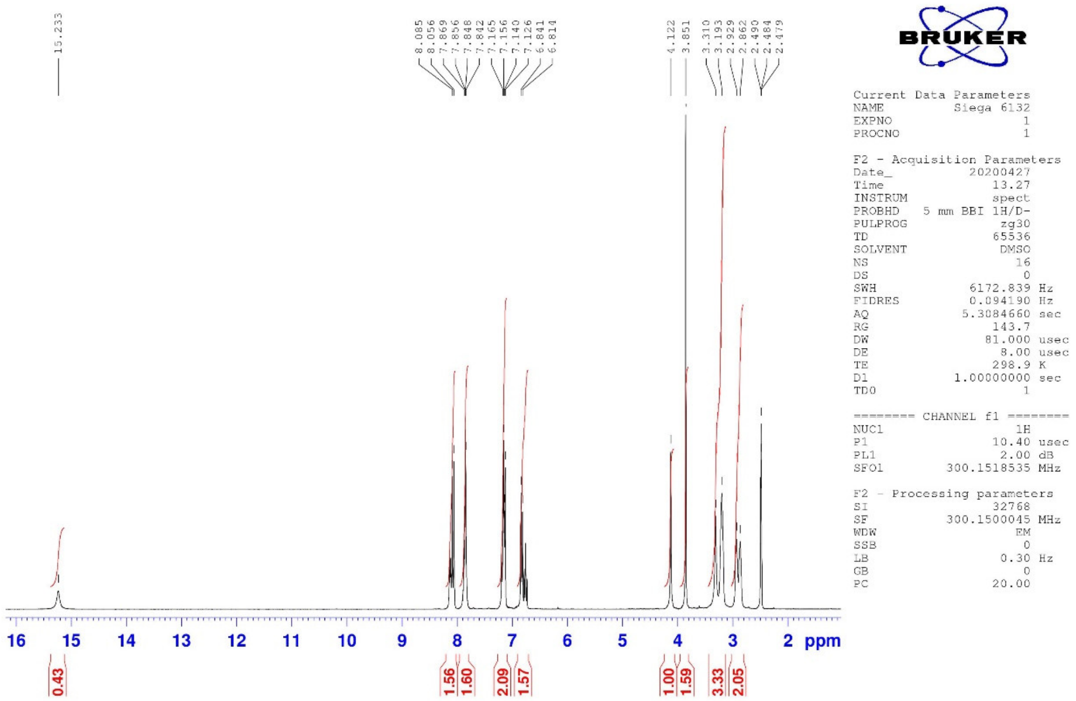

^{1}H NMR spectrum of **7j** in DMSO (300 MHz).

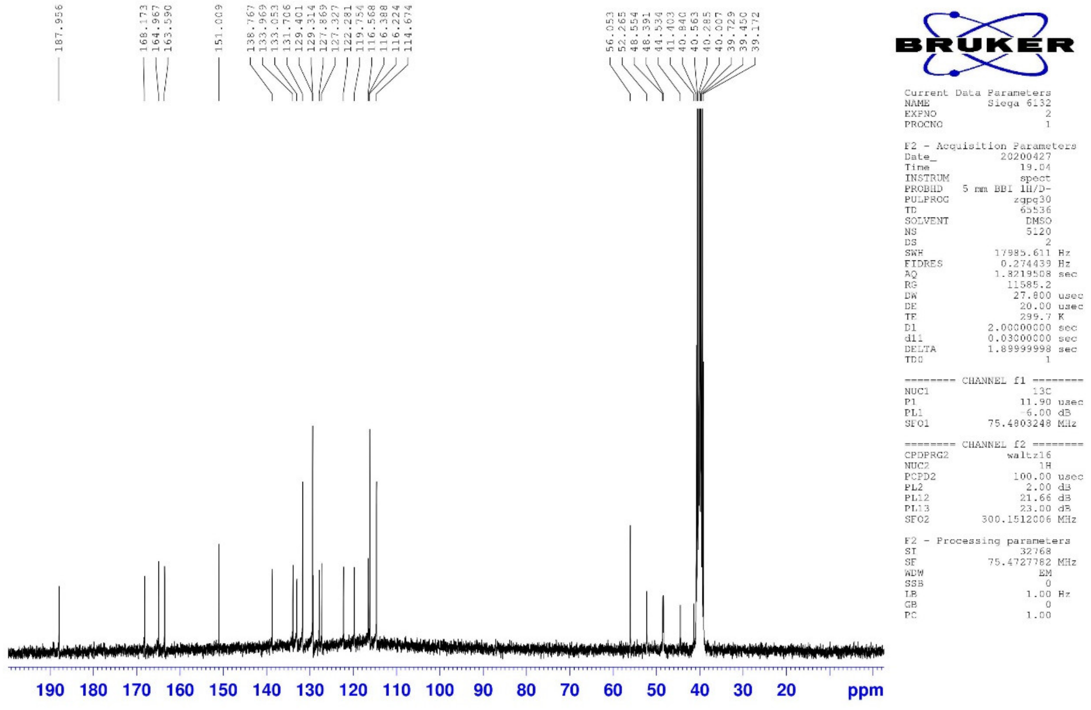

^{13}C NMR spectrum of **7j** in DMSO (300 MHz).

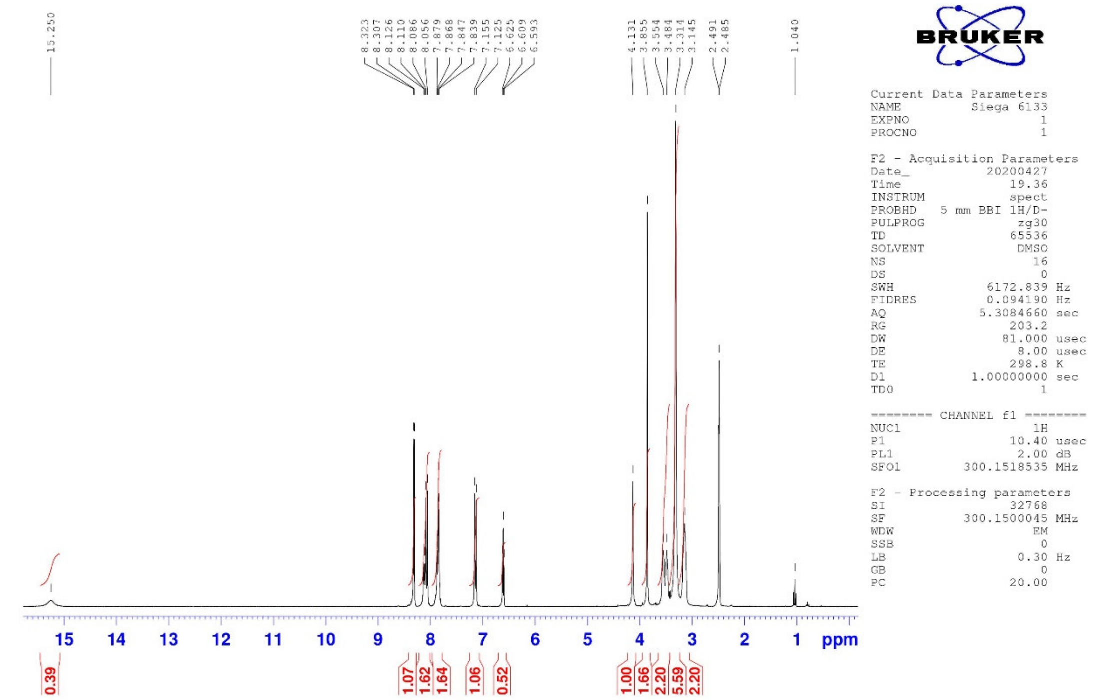

^{1}H NMR spectrum of **7k** in DMSO (300 MHz).

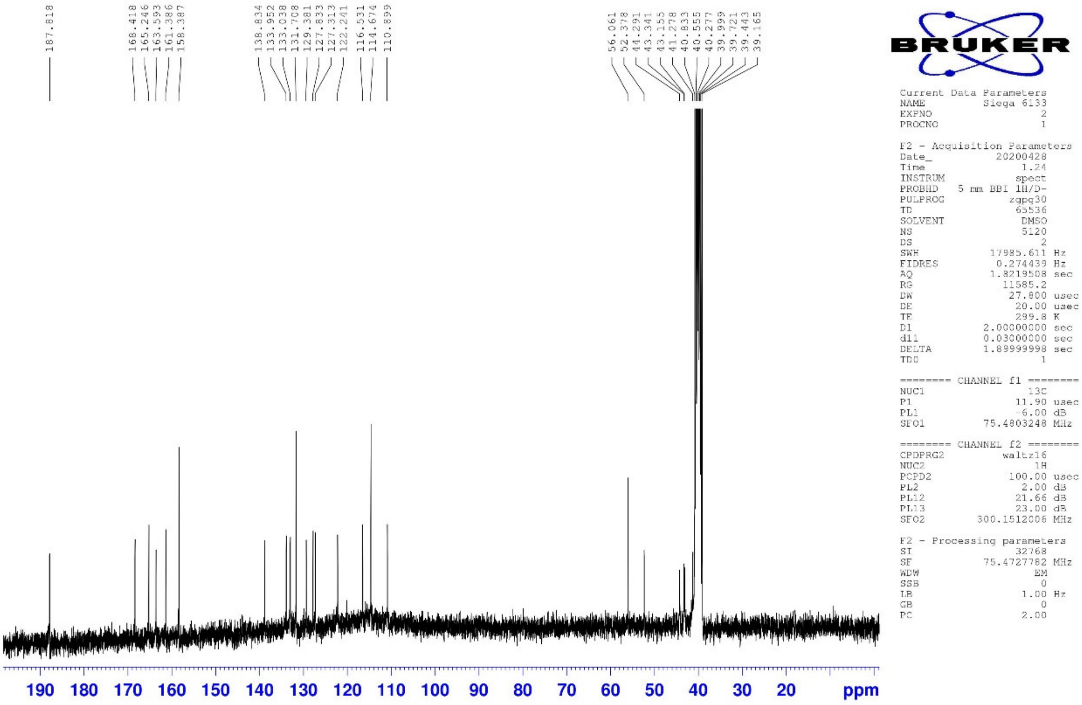

^{13}C NMR spectrum of **7k** in DMSO (300 MHz).

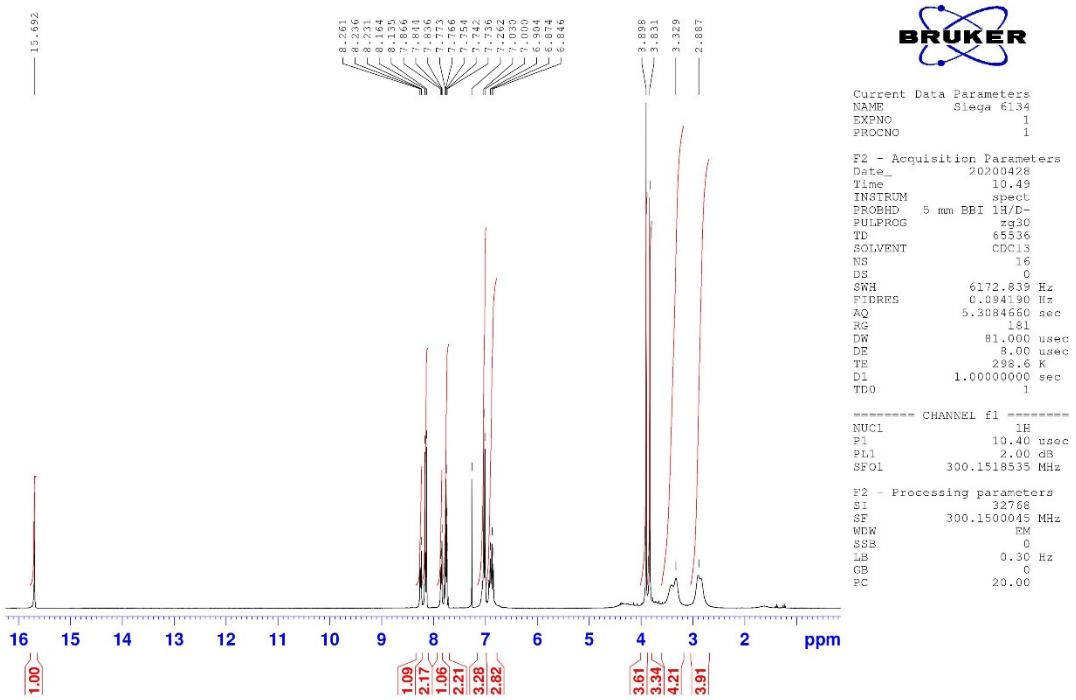

^{1}H NMR spectrum of **7l** in $CDCl_3$ (300 MHz).

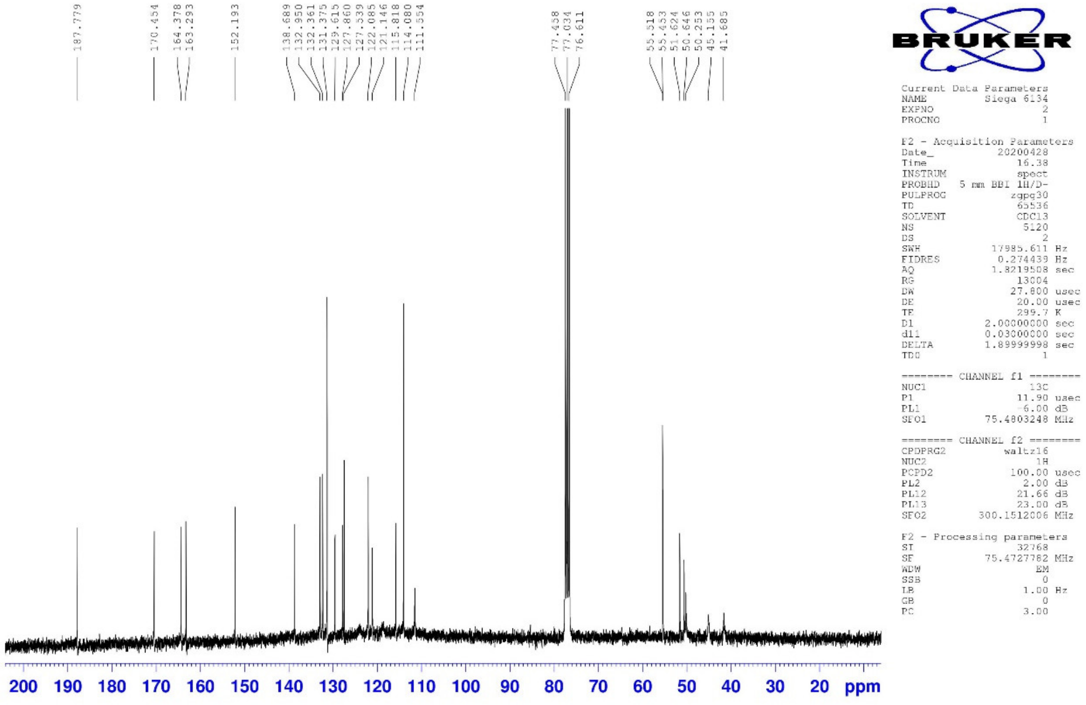

^{13}C NMR spectrum of **7l** in $CDCl_3$ (300 MHz)

Table S2. Inhibition zones [mm] of *S. aureus* growth being result of tested compounds' activity.

	Zones of *S. aureus* growth inhibition [mm]											
	4 mg	*2 mg*	*1 mg*	*0,5 mg*	*0,25 mg*	*0,2 mg*	*0,125 mg*	*0,1 mg*	*0,0625 mg*	*0,05 mg*	*0,0312 mg*	*0,025 mg*
**3e**	8 (±0)	8 (±0)	8	0	0	N/A	0	N/A	0	N/A	N/A	N/A
						**Series 6**						
**6a**	N/A	N/A	N/A	N/A	N/A	0	N/A	0	N/A	0	N/A	0
**6b**	0	0	0	0	0	N/A	0	N/A	8 (±0)	N/A	N/A	N/A
**6c**	0	0	0	0	0	N/A	0	N/A	0	N/A	N/A	N/A
**6d**	0	0	0	8 (±0)	8 (±0)	N/A	9 (±0)	N/A	9 (±0)	N/A	10 (±0)	N/A
**6e**	0	0	0	0	0	N/A	0	N/A	0	N/A	N/A	N/A
**6f**	0	0	0	0	0	N/A	0	N/A	0	N/A	N/A	N/A
**6g**	N/A	N/A	N/A	N/A	N/A	6 (±0)	N/A	6 (±0)	N/A	8 (±0)	N/A	9 (±0)
						**Series 7**						
**7a**	0	0	9,33 (±0,58)	9,66 (±1,15)	8,33 (±0,58)	N/A	9 (±1)	N/A	0	N/A	N/A	N/A
**7b**	10 (±0)	10 (±0)	10,33 (±0,58)	10 (±0)	9,33 (±0,58)	N/A	7,33 (±1,15)	N/A	0	N/A	N/A	N/A
**7c**	0	8 (±0)	9 (±0)	0	0	N/A	0	N/A	0	N/A	N/A	N/A
**7d**	0	0	0	0	8 (±0)	N/A	6 (±0)	N/A	N/A	N/A	N/A	N/A
**7e**	0	0	0	9 (±0)	9 (±0)	N/A	8 (±0)	N/A	6 (±0)	N/A	N/A	N/A
**7f**	0	0	0	9 (±0)	8 (±0)	N/A	9 (±0)	N/A	10 (±0)	N/A	10 (±0)	N/A
**7g**	0	0	0	0	6 (±0)	N/A	9 (±0)	N/A	8 (±0)	N/A	N/A	N/A
**7h**	0	10 (±0)	8 (±0)	9 (±0)	9 (±0)	N/A	0	N/A	0	N/A	N/A	N/A
**7i**	0	0	0	7 (±0)	7 (±0)	N/A	6 (±0)	N/A	6 (±0)	N/A	N/A	N/A
**7j**	0	0	0	0	0	N/A	0	N/A	0	N/A	N/A	N/A
**7k**	N/A	N/A	N/A	N/A	N/A	N/A	N/A	9 (±0)	N/A	6 (±0)	N/A	N/A
**7l**	0	0	9 (±0)	9 (±0)	8 (±0)	N/A	9 (±0)	N/A	0	N/A	N/A	N/A

N/A – Not applicable

Table S3. Inhibition zones [mm] of *E. faecalis* growth being result of tested compounds' activity.

	Zones of *E. faecalis* growth inhibition [mm]											
	4 mg	*2 mg*	*1 mg*	*0,5 mg*	*0,25 mg*	*0,2 mg*	*0,125 mg*	*0,1 mg*	*0,0625 mg*	*0,05 mg*	*0,0312 mg*	*0,025 mg*
**3e**	0	10 (±0)	10 (±0)	0	0	N/A	0	N/A	0	N/A	N/A	N/A
						**Series 6**						
**6a**	N/A	N/A	N/A	N/A	N/A	0	N/A	0	N/A	0	N/A	0
**6b**	0	0	0	0	0	N/A	10 (±0)	N/A	10 (±0)	N/A	N/A	N/A
**6c**	0	0	0	0	0	N/A	0	N/A	0	N/A	N/A	N/A
**6d**	0	0	0	10 (±0)	10 (±0)	N/A	11 (±0)	N/A	10 (±0)	N/A	10,33 (±0,58)	N/A
**6e**	0	0	0	0	0	N/A	0	N/A	0	N/A	N/A	N/A
**6f**	0	0	0	0	0	N/A	0	N/A	0	N/A	N/A	N/A
**6g**	N/A	N/A	N/A	N/A	N/A	0	N/A	0	N/A	0	N/A	0
						**Series 7**						
**7a**	0	0	0	9 (±0)	0	N/A	0	N/A	0	N/A	N/A	N/A
**7b**	0	0	0	9 (±0)	8 (±0)	N/A	8 (±0)	N/A	0	N/A	N/A	N/A

**7c**	0	0	0	0	0	N/A	0	N/A	0	N/A	N/A	N/A
**7d**	0	0	0	8 (±0)	7 (±0)	N/A	7 (±0)	N/A	N/A	N/A	N/A	N/A
**7e**	0	0	0	0	0	N/A	0	N/A	6 (±0)	N/A	N/A	N/A
**7f**	0	0	0	6 (±0)	8 (±0)	N/A	9 (±0)	N/A	11 (±0)	N/A	11,33 (±0,58)	N/A
**7g**	0	0	0	0	9 (±0)	N/A	10 (±0)	N/A	8 (±0)	N/A	N/A	N/A
**7h**	0	0	0	0	0	N/A	0	N/A	0	N/A	N/A	N/A
**7i**	0	0	0	0	0	N/A	0	N/A	6 (±0)	N/A	N/A	N/A
**7j**	0	0	0	9 (±0)	9 (±0)	N/A	9 (±0)	N/A	6 (±0)	N/A	N/A	N/A
**7k**	N/A	N/A	N/A	N/A	N/A	N/A	N/A	0	N/A	0	N/A	N/A
**7l**	0	0	0	0	0	N/A	0	N/A	0	N/A	N/A	N/A

N/A – Not applicable

Table S4. MIC and MBC values (mg/mL) for Gram-positive microorganisms. “X” – no bactericidal activity (MBC) in tested concentration of compound applied.

		6d		6g		7f	
	Microorganism	MIC	MBC	MIC	MBC	MIC	MBC
**G+**	*S. aureus*	0.024	0.1	0.00975	0.625	0.024	0.78
	E. faecalis	0.024	**X**	0.00975	**X**	0.024	**X**

Table S5. MIC and MBC values (mg/mL) for Gram-negative microorganisms. “X” – no bactericidal activity (MBC) in tested concentration of compound applied.

		7i		7l	
	Microorganism	MIC	MBC	MIC	MBC
**G-**	*P. aeruginosa*	1.56	3.125	1.56	3.125
	E. coli	1.56	3.125	3.125	3.125
	K. pneumoniae	1.56	3.125	3.125	3.125

Figure S1. Inhibition of bacterial growth in the area of precipitation zone.

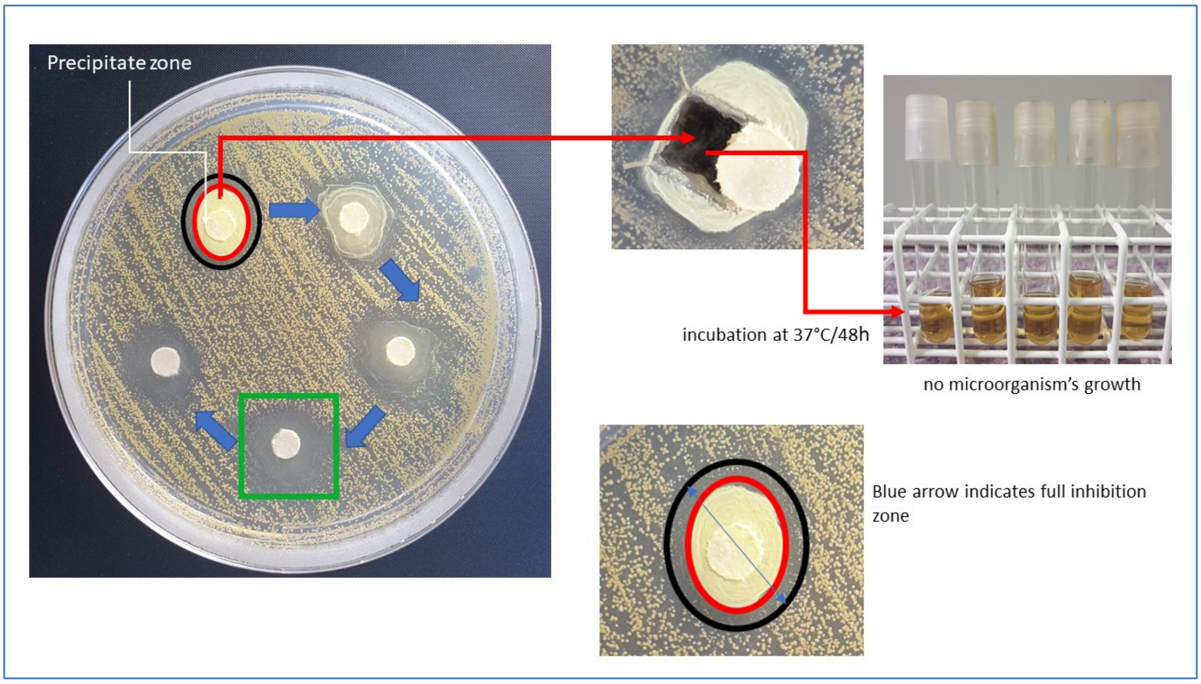

Figure S1: red circle – precipitant zone; black circle – halo zone. Thick blue arrows indicate decreasing quantities of compound 6d in a disc along with decreasing density of precipitant zone; the green square shows the concentration of 6d compound where no precipitant zone occurs. The right part of picture panel shows the removal of agar medium covered with precipitant and its introduction to sterile medium. No visible growth of microorganisms was observed during 48h of incubation proving the bactericidal activity of compound 6d within precipitant-covered area. This procedure was performed for all quantities of 6d compound presented in a Petri dish; the analogical results (microbial sterility) were obtained.

Table S6. Crystal data and details of the structure determination for compound **7i**

Formula	$C_{27}H_{24}FN_3O_5S$
Formula weight (g·mol-1)	521.55
Crystal system	orthorhombic
Space group	*F d d 2* (no. 43)
a (Å)	19.565(2)
b (Å)	48.341(5)
c (Å)	11.385(2)
V (Å3)	10768(2)
Z	16
Dcalc/Dobs (g·cm-1)	1.287/ 1.28
F000	4352
µ (mm-1)	0.168
Absorption correction	multi-scan
Tmin/Tmax	0.9757/ 1.000
Crystal size (mm)	0.38 × 0.36 × 0.21
T (K)	295(2)
Radiation type/wavelength (Å)	Mo K*α* / 0.71073
◎ range (°)	2.679-25.493
No. of measured refections	30669
No. of independent refections	4816

Rint	0.0538
No. of refns with I > 2σ(I)	3132
No. of parameters/restraints	328/3
R1 [I > 2 σ(I)][a]	0.0538
wR2 (all data)[a]	0.0988
S	1.01
Δρmax/Δρmin (e Å^3)	+0.245/-0.212

[a] $R_1 = \Sigma||F_o| - |F_c||/\Sigma|Fo|$, $wR_2 = \{\Sigma [w(F_o^2 - F_c^2)^2]/ \Sigma[w(F_o^2)^2]\}^{½}$, where $w^{-1}=[\sigma^2(F_o^2) + (0.0392P)^2]$, where $P = (F_o^2 + 2F_c^2)/3$.

Table S7. The highest tested concentration 1,2-benzothiazine 1,1-dioxide derivatives

Compound	The highest concentration applied [mg/ml]	Applied concentration of DMSO [%}
**3e**	33.33	50
**6a**	5	50
**6b**	12.5	50
**6c**	8.3	50
**6d**	12.5	50
**6e**	12.5	50
**6f**	12.5	50
**6g**	5	50
**7a**	16.65	50
**7b**	25	50
**7c**	8.3	50
**7d**	12.5	50
**7e**	2.1	50
**7f**	12.5	50
**7g**	12.5	50
**7h**	12.5	50
**7i**	50	50
**7j**	12.5	50
**7k**	2.5	50
**7l**	25	50

Cytotoxicity data

Table S8. The absorbance values of samples and control.

Number of compound and tested concentration [mg/mL]	Absorption (540nm)			SEM	Positive control of fibroblasts' growth
**3e (1,04)**	0.0970	0.1319	0.1802	0.041779	0.3296; 0.3272;
**3e (2,08)**	0.1181	0.1292	0.0934	0.018325	0.2731;
**6a (0,078)**	0.3221	0.3562	0.3485	0.017884	0.2812; 0.310;5
**6b (0,024)**	0.2827	0.2854	0.2906	0.004015	0.3257;

**6c (0,024)**	0.2543	0.302	0.2973	0.026288	0.288; 0.3066; 0.2945; 0.3396; 0.3214; 0.2934; 0.2942; 0.2959; 0.3344; 0.3119; 0.2732; 0.2974; 0.3129; 0.3132; 0.3214; 0.2815; 0.3061; 0.2949; 0.422; 0.404;1 0.4077; 0.4526; 0.4102; 0.4142; 0.4184; 0.4134; 0.4562; (μ=0.33717; ±0.05605)
**6d (0,024)**	0.0666	0.0706	0.0675	0.002098	
**6e (0,024)**	0.2669	0.2373	0.2685	0.01757	
**6f (0,195)**	0.2963	0.2839	0.2786	0.009084	
**6g (0,00975)**	0.2472	0.2886	0.2608	0.021102	
**7a (0,26)**	0.2823	0.3233	0.3817	0.049953	
**7a (0,065)**	0.123	0.3273	0.3491	0.124723	
**7b (0,195)**	0.3613	0.3298	0.3588	0.01751	
**7b (0,39)**	0.3991	0.369	0.353	0.023407	
**7c (0,13)**	0.3400	0.3454	0.3496	0.004812	
**7d (0,024)**	0.3811	0.3679	0.3747	0.006601	
**7e (0,655)**	0.3214	0.3186	0.3444	0.014157	
**7f (0,024)**	0.2891	0.3156	0.3773	0.045256	
**7g (0,024)**	0.2886	0.3128	0.3122	0.013802	
**7h (0,78)**	0.1801	0.2496	0.1948	0.036627	
**7i (0,78)**	0.1114	0.1149	0.1217	0.005237	
**7i (1,56)**	0.1344	0.143	0.1106	0.016784	
**7j (0,195)**	0.2993	0.3293	0.3394	0.020857	
**7j (0,39)**	0.1487	0.281	0.2528	0.069684	
**7k (0,156)**	0.245	0.2591	0.3327	0.047094	
**7l (0,195)**	0.3301	0.307	0.3147	0.011762	
**7l (0,39)**	0.3334	0.3285	0.3182	0.007758	

Table S9. Concentrations of DMSO [%] used for 1,2-benzothiazine 1,1-dioxide derivatives dissolution to perform cytotoxicity assay.

Compound	Concentrations [mg/ml]	Concentration of DMSO [%]
**3e**	1.04*/2.08**	1.562*/3.125**
**6a**	0.078	0.781
**6b**	0.024	0.098
**6c**	0.065	0.39
**6d**	0.024	0.098
**6e**	0.024	0.098
**6f**	0.195	0.781
**6g**	0.00975	0.098
**7a**	0.26*/0.065**	0.781*/0.195**
**7b**	0.195*/0.39**	0.390*/0.781**
**7c**	0.13	0.781
**7d**	0.024	0.098
**7e**	0.655	1.562
**7f**	0.024	0.098
**7g**	0.024	0.098
**7h**	0.78	3.125
**7i**	0.78*/1.56**	0.781*/1.562**
**7j**	0.195*/0.39**	0.781*/1.562**
**7k**	0.156	3.125
**7l**	0.195*/0.39**	0.390*/0.781**

* ; ** : one or two asterisks are assigned to the specific compound concentration/specific DMSO concentration applied.

Table S10. Cytotoxicity of applied concentrations of DMSO. The cytotoxicity was calculated as % ($\%C_{tx}$) using the formula: $\%C_{tx}$=100%- (A_b/A_k)*100%
A_b=Absorbance of the sample
A_k=Absorbance of control

Concentration of DMSO [%]	Absorption (540nm)			SEM	Positive control of fibroblasts' growth	Cytotoxycity [%]
**0.024414063**	0.2959	0.3586	0.3272	0.0314	0.3296; 0.3272; 0.2731; 0.2812; 0.310;5 0.3257; 0.288; 0.3066; 0.2945; 0.3396; 0.3214; 0.2934; 0.2942; 0.2959; 0.3344; 0.3119; 0.2732; 0.2974; 0.3129; 0.3132; 0.3214; 0.2815; 0.3061; 0.2949; 0.422; 0.404;1 0.4077; 0.4526; 0.4102; 0.4142; 0.4184; 0.4134; 0.4562.(μ=0.33717)	**2.946992**
**0.048828125**	0.3184	0.325	0.346	0.0144		**2.185753**
**0.09765625**	0.3513	0.3447	0.3588	0.0071		**-4.27983**
**0.1953125**	0.3016	0.3523	0.3804	0.0399		**-2.25316**
**0.390625**	0.3323	0.3085	0.3769	0.0347		**-0.61205**
**0.78125**	0.3224	0.3365	0.3901	0.0357		**-3.70643**
**1.5625**	0.3514	0.3618	0.3694	0.0090		**-7.0282**
**3.125**	0.2815	0.3605	0.3684	0.0481		**0.109647**
**6.25**	0.0952	0.1461	0.1138	0.0258		**64.89404**
**12.5**	0.0622	0.0703	0.0559	0.0072		**81.37436**
**25**	0.0619	0.0602	0.0595	0.0012		**82.04663**
**50**	0.0669	0.0592	0.0544	0.0063		**82.15538**
